# Therapeutic potential of targeting MKK3-p38 axis with Capsaicin for Nasopharyngeal Carcinoma

**DOI:** 10.7150/thno.45191

**Published:** 2020-06-24

**Authors:** Chengyao Chiang, Min Zhang, Dian Wang, Tian Xiao, Lizhi Zhu, Kai Chen, Junrong Huang, Jingying Huang, Jiang Zhu, Li Li, Cheng Chen, Yangchao Chen, Hongyi Hu, Wenqi Jiang, Yongdong Zou, Ting Wang, Duo Zheng

**Affiliations:** 1 Guangdong Provincial Key Laboratory of Regional Immunity and Diseases, Shenzhen University International Cancer Center, Department of Cell Biology and Genetics, School of Medicine, College of Life Sciences and Oceanography, Shenzhen University, Shenzhen 518055, China.; 2 Xiangya School of Pharmaceutical Sciences, Central South University, Changsha, 410013, China.; 3 Institute of Translation Medicine, Shenzhen Second People's Hospital, The First Affiliated Hospital of Shenzhen University, Shenzhen, 518035, China.; 4 School of Materials Science and Engineering, Central South University of Forestry and Technology, Changsha, 410004, China.; 5 School of Biomedical Sciences, Faculty of Medicine, The Chinese University of Hong Kong, Shatin, NT, Hong Kong.; 6 Department of Otolaryngology, Peking University Shenzhen Hospital, Shenzhen, 518036, China.; 7 Sun Yet-sen University Cancer Center, Guangzhou, China.; 8 Guangzhou Henvcom Bioscience Co Ltd, Guangzhou, 510535, China.

**Keywords:** Capsaicin, MKK3-p38, nasopharyngeal carcinoma, FUK, cell mobility

## Abstract

**Background:** Capsaicin is an active compound found in plants of the *Capsicum* genus; it has a range of therapeutic benefits, including anti-tumor effects. Here we aimed to delineate the inhibitory effects of capsaicin on nasopharyngeal carcinoma (NPC).

**Methods:** The anti-cancer effects of capsaicin were confirmed in NPC cell lines and xenograft mouse models, using CCK-8, clonogenic, wound-healing, transwell migration and invasion assays. Co-immunoprecipitation, western blotting and pull-down assays were used to determine the effects of capsaicin on the MKK3-p38 axis. Cell proliferation and EMT marker expression were monitored in MKK3 knockdown (KD) or over-expression NPC cell lines treated with or without capsaicin. Finally, immunohistochemistry was performed on NPC specimens from NPC patients (n = 132) and the clinical relevance was analyzed.

**Results:** Capsaicin inhibited cell proliferation, mobility and promoted apoptosis in NPC cells. Then we found that capsaicin directly targets p38 for dephosphorylation. As such, MKK3-induced p38 activation was inhibited by capsaicin. Furthermore, we found that capsaicin-induced inhibition of cell motility was mediated by fucokinase. Xenograft models demonstrated the inhibitory effects of capsaicin treatment on NPC tumor growth *in vivo*, and analysis of clinical NPC samples confirmed that MKK3 phosphorylation was associated with NPC tumor growth and lymphoid node metastasis.

**Conclusions:** The MKK3-p38 axis represents a potential therapeutic target for capsaicin. MKK3 phosphorylation might serve as a biomarker to identify NPC patients most likely to benefit from adjunctive capsaicin treatment.

## Introduction

Nasopharyngeal carcinoma (NPC) is a malignant epithelial tumor of the nasopharynx that has a unique, geographically-influenced incidence rate; it is particularly common in Southeast Asia, Alaska and Greenland 1-3. Certain ethnic groups are disproportionately affected by NPC. For example, in a high-risk area of South China, men and women experience incidence rates of 27.2/100,000 and 11.3/100,000, respectively 4, 5. Conversely, the incidence rate is <1/100,000 in most other populations 1. NPC is a polygenic disease with several well-known environmental risk factors, which include infection with the Epstein-Barr virus (EBV) 6, consumption of salted fish, smoking and exposure to wood dust 7. Certain single nucleotide polymorphisms (SNPs) are reportedly associated with NPC development and progression, including SNPs in the *HLA-A, -B, -C, -DQ/DR, TNFRSF19, MDS1-EVI1, CDKN2A/B* loci 8. The prevalence of such SNPs in different ethnic groups might explain why certain populations are at a higher risk of developing NPC than others.

NPC is invasive and highly metastatic 9-11. The preferred treatment approach primarily depends on the tumor-node-metastasis (TNM) staging category, with patients with early-stage NPC receiving radiotherapy and those with advanced NPC receiving chemoradiotherapy 12, 13. This combined-modality therapy has increased the NPC 5-year survival rates from 61% to 73%, but the distant metastasis rate of NPC in the advanced stages remains as high as 30% 12. Although NPC is sensitive to radiotherapy, ~30% of NPC patients fail to respond to treatment and go on to develop local recurrence and distant metastasis 14, 15. Unfortunately, the causes underlying treatment failure remain unclear; therefore, the identification of novel tumor markers and therapeutic targets for patients with advanced NPC is of the utmost importance.

The pharmacological and physiological effects of capsaicin, an active component of chili peppers, have been investigated in the context of a broad range of conditions 16. The compound has cardioprotective properties 17 and is known to have anti-inflammatory 18, analgesic 19, antioxidant 20 and anti-obesity 21 effects. Furthermore, capsaicin can relieve pain in patients with arthritis, postoperative neuralgia, diabetic neuralgia and psoriasis 22. However, the effect of capsaicin on cancer is somewhat controversial, and the underlying molecular mechanisms are unclear. For example, previous epidemiological studies have shown that excessive capsaicin uptake might increase the risk of gastrointestinal carcinogenesis 23. However, capsaicin also seems to suppress cell growth in both gastric 24, 25 and bladder cancer 26 by inhibiting cell survival signaling pathways in immortalized cell lines. Furthermore, capsaicin-induced cell cycle arrest has been reported in breast cancer 27 and colorectal cancer 28. In terms of the underlying molecular mechanisms, capsaicin triggers apoptosis through endoplasmic reticulum stress 29 and by downregulating the PI3K-Akt axis in NPC 30. Finally, capsaicin inhibits p38 phosphorylation to restrain cell invasion and metastasis in fibrosarcoma 31.

The effect of capsaicin on the p38 signaling pathway is of particular interest, as this pathway is critical to cancer progression and metastasis 32-34. MKK3 and MKK6 are kinases upstream of p38, and are involved in cell differentiation, division, migration, apoptosis and stress responses 35. Activated p38 regulates various transcription factors and thus the expression of many downstream genes. The MKK3-p38 axis in particular seems to regulate tumor invasion 36, 37 and progression 38.

Here, we aimed to investigate the molecular mechanisms underlying the tumor-inhibiting effects of capsaicin in NPC. We decided to focus on the potential involvement of the p38 signaling pathway. First, we confirmed the anti-cancer effects of capsaicin treatment in NPC, and then investigated the significance of the MKK3-p38 axis to NPC development and progression *in vitro, in vivo* and in patient samples. We found that capsaicin inhibits MKK3-induced p38 activation by directly targeting p38. We also found that fucose kinase (FUK), an inhibitor of metastasis regulated by ATF2 and a transcription factor downstream of p38 39, regulates the anti-cancer effects of capsaicin. The MKK3-p38 axis might represent a novel target for NPC treatment: synergistic co-treatments involving capsaicin and other anti-cancer agents might have therapeutic potential in the future.

## Results

### Capsaicin inhibits NPC development and progression, and promotes apoptosis

**Figure 1:**
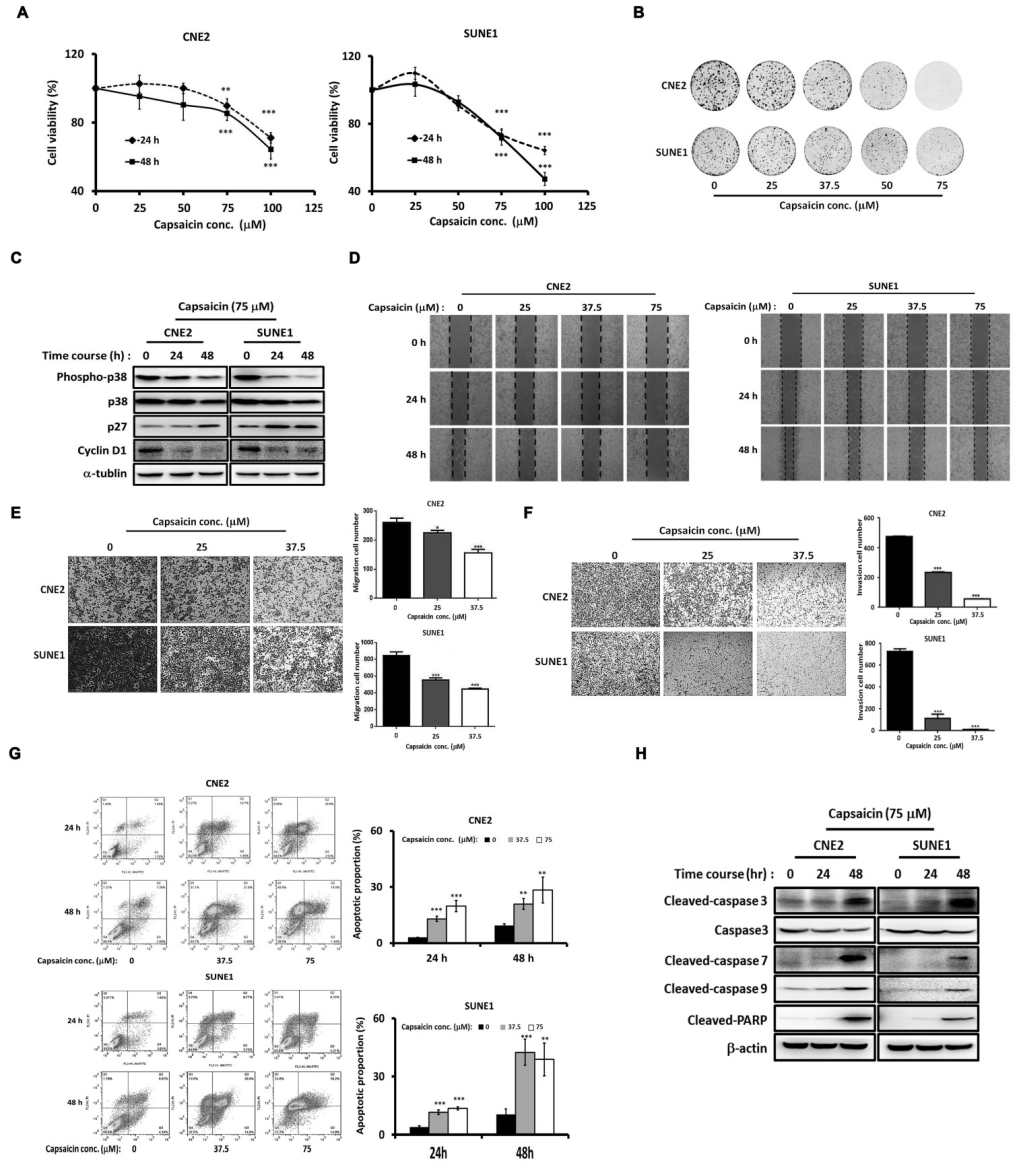
** Capsaicin-induced inhibition of NPC progression is dose-dependent.** (A) CNE2 and SUNE1 cell growth was inhibited by capsaicin treatment. (B) The inhibitory effect of capsaicin was demonstrated by clonogenic assay in CNE2 and SUNE1 cells. (C) The expression of cell cycle-associated molecules was altered in CNE2 and SUNE1 cells following capsaicin treatment. (D) Capsaicin suppressed the migratory capacities of CNE2 and SUNE1 cells. (E) Transwell assays were used to test the migratory capacity of CNE2 and SUNE1 following capsaicin treatment. (F) The invasive abilities of CNE2 and SUNE1 were tested following capsaicin treatment. (G) Apoptosis was induced in CNE2 and SUNE1 cells by capsaicin treatment. Annexin V-fluorescein isothiocyanate and propidium iodide staining were used to label early- and late-apoptotic cells, respectively. (H) The protein expression of pro-apoptotic markers was increased following capsaicin treatment. The data represent the means ± standard deviation. *p<0.05; **p<0.01; ***p<0.0001.

Previous studies have shown the anticancer effects of capsaicin in NPC 29, 30. To investigate the molecular mechanisms involved, we first confirmed the anticancer efficacy of capsaicin in CNE2 and SUNE1 NPC cell lines. We found that CNE2 and SUNE1 cell growth was inhibited by capsaicin in a dose-dependent manner (Figure 1A). Clonogenic assays further confirmed a dose-dependent capsaicin-induced inhibition of cell viability (Figure 1B). Furthermore, the expression of cell cycle-associated molecules was altered by capsaicin treatment in both cell lines (Figure 1C). For example the cell-cycle inhibitor p27^Kip1^ was upregulated after capsaicin treatment while the G1-S phase transition marker cyclin D1 was downregulated. p38 phosphorylation levels were also decreased following capsaicin treatment (Figure 1C). Consistently, the mRNA expression of p38 downstream genes, including p27, p21, cyclin D1 and D2, were also down-regulated by capsaicin in NPC cells (Figure S1).

The results of a wound healing assay revealed that CNE2 and SNE1 cell migration was suppressed in a dose-dependent manner after capsaicin treatment for either 24 or 48 h (Figure 1D). Meanwhile, a Transwell assay revealed that CNE2 and SUNE1 migration was also inhibited by capsaicin, once again in a dose-dependent manner (Figure 1E). The same was true for cell invasion, as assessed using Matrigel (Figure 1F). Capsaicin was originally identified as a TRPV1 agonist 40. We thus treated the NPC cells with capsazepine (CPZ), a common TRPV1 antagonist 41. Interestingly, the inhibitory effects of capsaicin on cell migration and growth were not reversed by CPZ pretreatment, implying that capsaicin exerts its effects on NPC in a TRPV1-independent manner (Figure S2).

To assess the effects of capsaicin on apoptosis, we treated CNE2 and SUNE1 cells with capsaicin for 24 or 48 h, and found that apoptosis was induced in a dose-dependent manner. We confirmed these findings by flow cytometry (Figure 1G). Consistently, the expression levels of cleaved-caspase 3, 7, 9 and PARP (markers of apoptosis) were upregulated following capsaicin treatment (Figure 1H). Taken together, these results demonstrate that capsaicin has inhibitory effects on the growth, migration, invasion and survival of NPC cell lines. Inhibiting the p38 signaling pathway might underlie the anti-cancer effects of capsaicin.

### Capsaicin interrupts the MKK3-p38 pathway and targets p38

**Figure 2:**
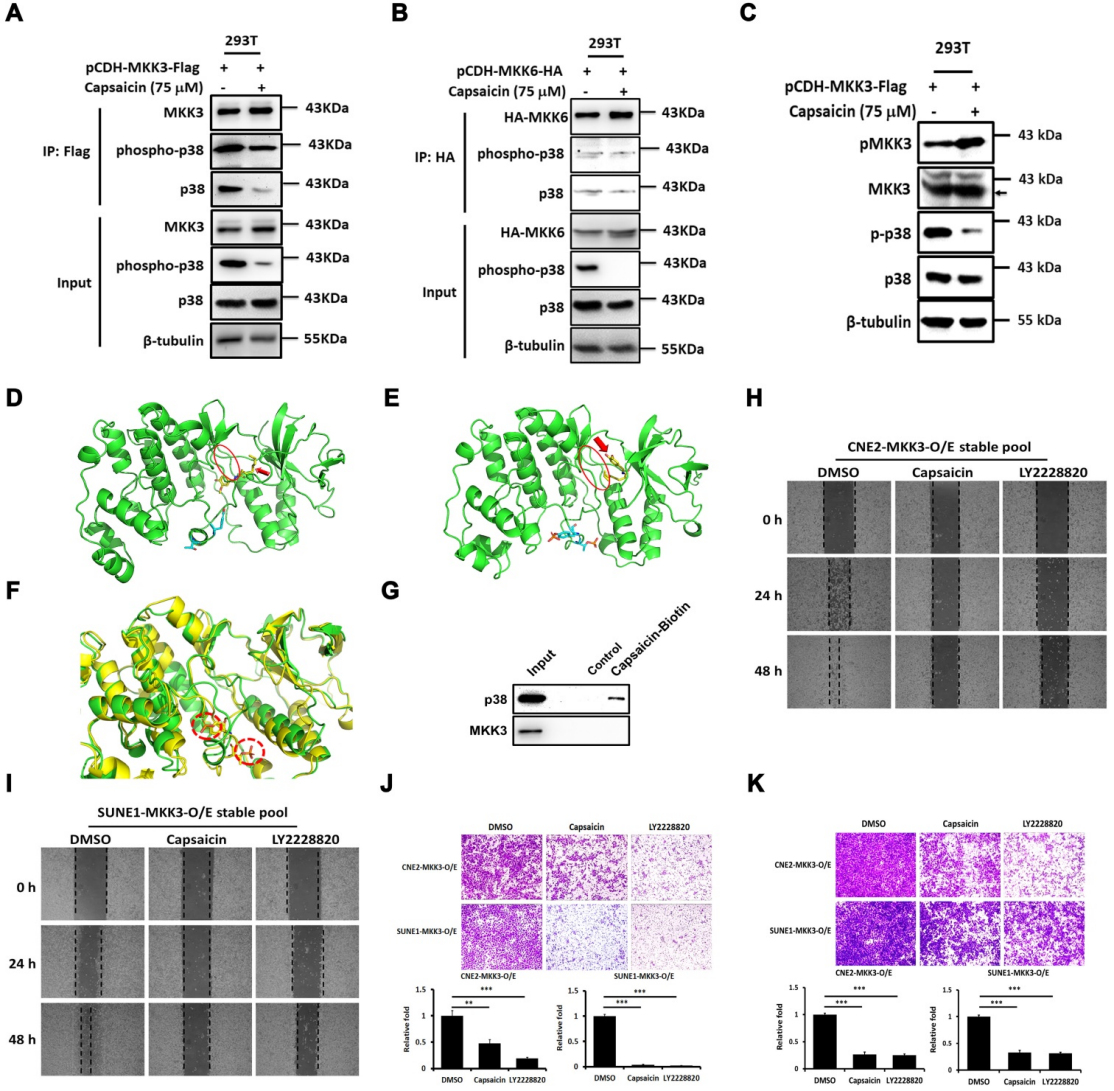
** Capsaicin directly targets p38 and blocks the interaction between MKK3 and p38.** Co-immunoprecipitation of p38 was performed in HEK293T cells overexpressing MKK3 or MKK6. (A and B) Capsaicin treatment (75 μM) inhibited the interaction between p38 and MKK3 (A) but not the interaction between p38 and MKK6 (B). (C) MKK3-induced p38 phosphorylation was blocked by capsaicin treatment in HEK293T cells. (D and E) Computer modeling analysis revealed that capsaicin bound into the pocket structure of p38. The kinetic free energy of capsaicin binding with unphosphorylated p38 was -12.3 kcal/mol (D) and with phosphorylated p38 was -6.2 kcal/mol (E). Red oval, the loop of the pocket where capsaicin binds; red arrow, capsaicin. (F) Superposed structures of phosphorylated and unphosphorylated p38 showing that the loop in the bottom of the pocket is affected by the insertion of capsaicin. The loop conformations near the pocket changes due to p38 phosphorylation. Red-dotted circles, phosphorylated p38 residues. (G) Pull-down assay showing that p38 directly binds capsaicin. (H and I) Cell migration was inhibited in CNE2 (H) and SUNE1 MKK3-overexpression stable pools (I) after capsaicin treatment (75 μM), and to a similar degree after LY2228820 treatment (10 μM). (J and K) Transwell assays revealed that the migration (J) and invasion (K) of CNE2 and SUNE1 MKK3-O/E stable pools was suppressed by either capsaicin (75 μM) or LY2228820 treatment (10 μM). The data represent the means ± standard deviation. **p<0.01; ***p<0.001.

We next aimed to clarify the molecular mechanism by which capsaicin exerts its anti-cancer effects, focusing on p38 signaling pathway. We performed immunoprecipitation experiments in HEK293T cells overexpressing MKK3 or MKK6, and found that the interaction between p38/phospho-p38 and MKK3 only was suppressed by capsaicin treatment (Figure 2A and 2B). Thus, p38 phosphorylation seemed to be primarily under the control of MKK3, with p38 phosphorylation being downregulated by capsaicin treatment even when MKK3 was overexpressed (Figure 2C).

Computer modeling analyses found that the free energy of capsaicin kinetically bound into the pocket of unphosphorylated-p38 (-12.3 kcal/mol; Figure 2D) was lower than that of capsaicin bound to phosphorylated-p38 (-6.2 kcal/mol; Figure 2E) or MKK3/6 (-6.4 and -5.6 kcal/mol, respectively; Figure S3). Superposed structures of phosphorylated- and unphosphorylated-p38 showed that a loop in the bottom of the pocket affected capsaicin binding. The loop conformations near the pocket changed due to phosphorylation (Figure 2F), causing a decrease in the binding affinity of capsaicin. This finding confirmed the greater affinity of capsaicin for unphosphorylated-p38 than either phosphorylated-p38 or MKK3/6. Importantly, a pull-down assay also revealed that p38, but not MKK3, was captured by capsaicin-conjugated beads, suggesting that capsaicin targets p38 directly (Figure 2G).

We next performed computer simulations to determine how capsaicin analogues, Zucapsaicin, Nonivamide and Capsazepine dock into the p38 pocket. The binding free energies of Nicoboxil, Capsazepine, and Zucapsaicin with p38 were -6.4, -10.5 and -8.9 kcal/mol, indicating that Capsaicin (-12.3 kcal/mol) has the highest potency among these ligands (Figure S4). Together, these results suggest that capsaicin binds with p38 to block MKK3-mediated p38 phosphorylation, leading to less phospho-p38 and inhibition of downstream signaling pathways.

Wound healing assays revealed that MKK3-induced CNE2 and SUNE1 cell migration was suppressed upon either capsaicin treatment (37.5 μM), p38 inhibitor treatment (LY2228820; 10 μM) or p38β shRNA (Figure 2H and I, Figure S5C and H). The MKK3-induced migratory capacity was also suppressed by capsaicin (37.5 μM), LY2228820 (10 μM) or p38β shRNA treatment in CNE2 and SUNE1 cells (Figure 2J, Figure S5D and I), and the same was true for MKK3-induced invasion (Figure 2K, Figure S5E and J). These results suggest that MKK3-p38 signaling is blocked by capsaicin treatment, and that p38 is the primary capsaicin target in this pathway.

### The MKK3-p38 axis is involved in NPC development and progression

**Figure 3:**
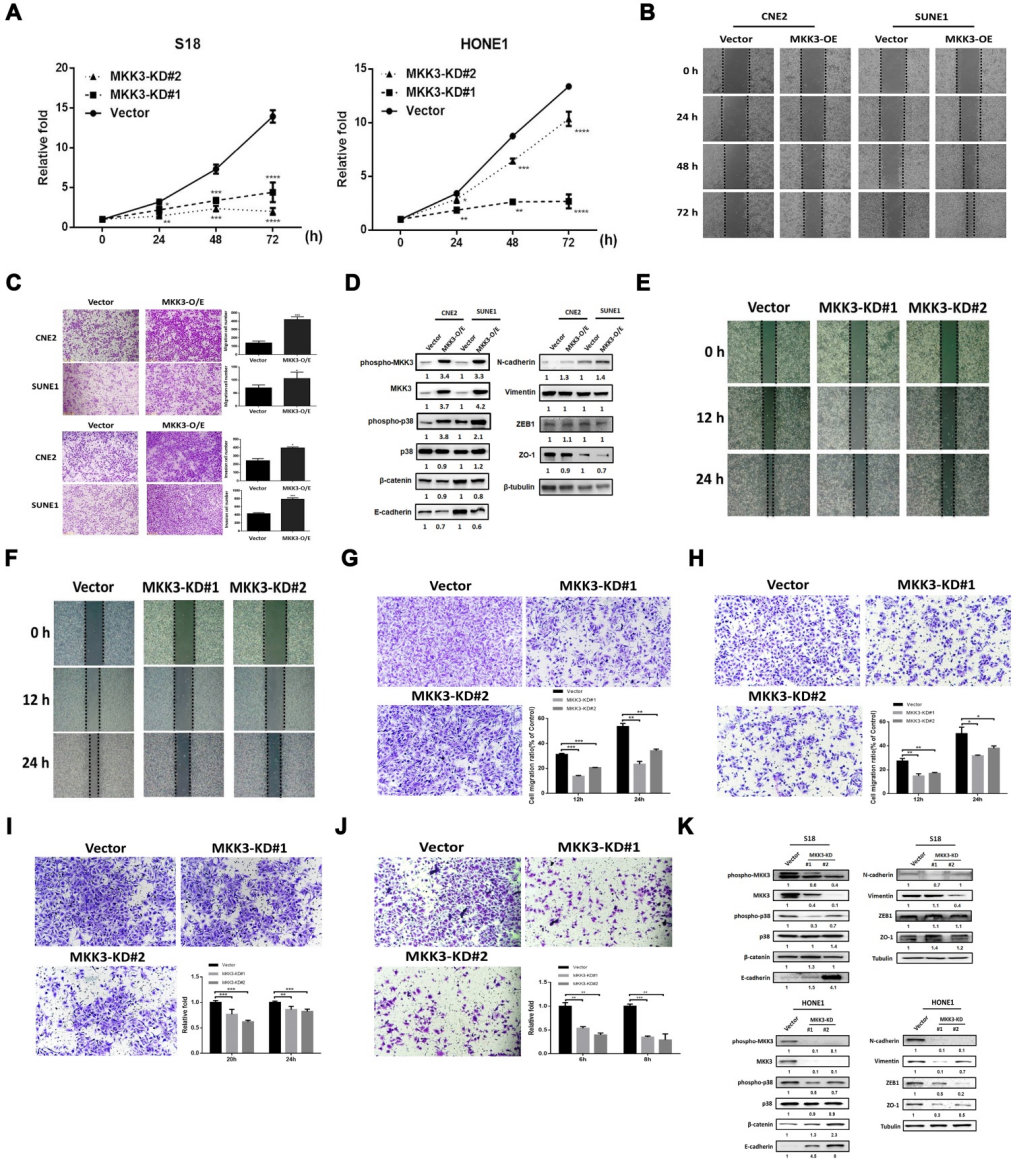
** The MKK3-p38 axis promotes tumor progression in nasopharyngeal carcinoma cell lines.** (A) Cell growth was suppressed in S18 and HONE1 MKK3-knockdown stable pools. (B and C) MKK3-overexpression promoted cell migration and invasion, as determined through wound healing (B) and Transwell and Matrigel assays (C). (D) MKK3 overexpression in CNE2 and SUNE1 cells resulted in the downregulation of the epithelial markers β-catenin, E-cadherin and ZO-1, and the upregulation of the mesenchymal markers vimentin and N-cadherin. ZEB-1 expression was unaffected. p38 and MKK3 were also upregulated following MKK3-overexpression. (E and F) MKK3 knockdown was suppressed cell migration in S18 (E) and HONE1 cells (F), as shown by wound healing assay. (G and H) MKK3 knockdown resulted in reduced migratory capabilities in S18 (G) and HONE1 cells (H), as assessed by Transwell assay. (I and J) Matrigel assays were used to assess invasion in S18 (I) and HONE1 MKK3-knockdown cells (J), and a similar effect was observed. (K) MKK3 knockdown resulted in the upregulation epithelial markers and the downregulation of mesenchymal markers, as well as reducing phosphorylation levels of p38 and MKK3. The data represent the means ± standard deviation. *p<0.05; **p<0.01; ***p<0.001.

MKK3 is known to be involved in tumor progression 36-38, but whether or how MKK3 signaling regulates NPC is elusive. We first screened MKK3 mRNA expression levels in NPC cell lines by qPCR (Figure S6). In addition, a CCK-8 assay showed that transient MKK3 knockdown in S18 and HONE1 (Figure S7 A, B, D and E) cells resulted in inhibited cell growth (Figure 3A). However, a promotion of cell growth was not observed when we overexpressed MKK3 in CNE2 and SUNE1 cells (Figure S8). Wound healing was enhanced by MKK3 overexpression in CNE2 and SUNE1 cells (Figure 3B); as were cell migration and invasion, as determined by Transwell assay (Figure 3C).

Western blot analysis revealed that MKK3 overexpression in SUNE1 and CNE2 promoted the phosphorylation of both MKK3 and p38 (Figure 3D). Furthermore, the epithelial markers β-catenin, E-cadherin and ZO-1 were downregulated by MKK3 overexpression, while the mesenchymal marker N-cadherin was upregulated. However, expression of vimentin and ZEB1, which are also used as mesenchymal markers, did not markedly change (Figure 3D).

Wound healing assays showed that S18 and HONE1 cell migration was inhibited by MKK3 knockdown (Figure 3E and F); we confirmed this finding and the inhibition of cell migratory and invasion by Transwell assay (Figure 3G-J). Furthermore, we found that the cell growth of stable MKK3-knockdown S18 and HONE1 cells did not significantly differ from that of control cells exposed cultured under the same conditions (2% FBS; Figure S7 C and F). Western blot analysis revealed that MKK3 and p38 phosphorylation decreased after MKK3 knockdown in S18 and HONE1 cells. β-catenin and E-cadherin protein expression was upregulated by MKK3 knockdown, while N-cadherin, vimentin and ZEB1 protein expression was downregulated. However, there were inconsistencies in terms of ZO-1 expression between the two cell lines (Figure 3K). These results indicate that the MKK3-p38 axis is a critical pathway associated with EMT in NPC progression.

### MKK3-p38 axis inhibition by capsaicin is mediated by FUK

**Figure 4:**
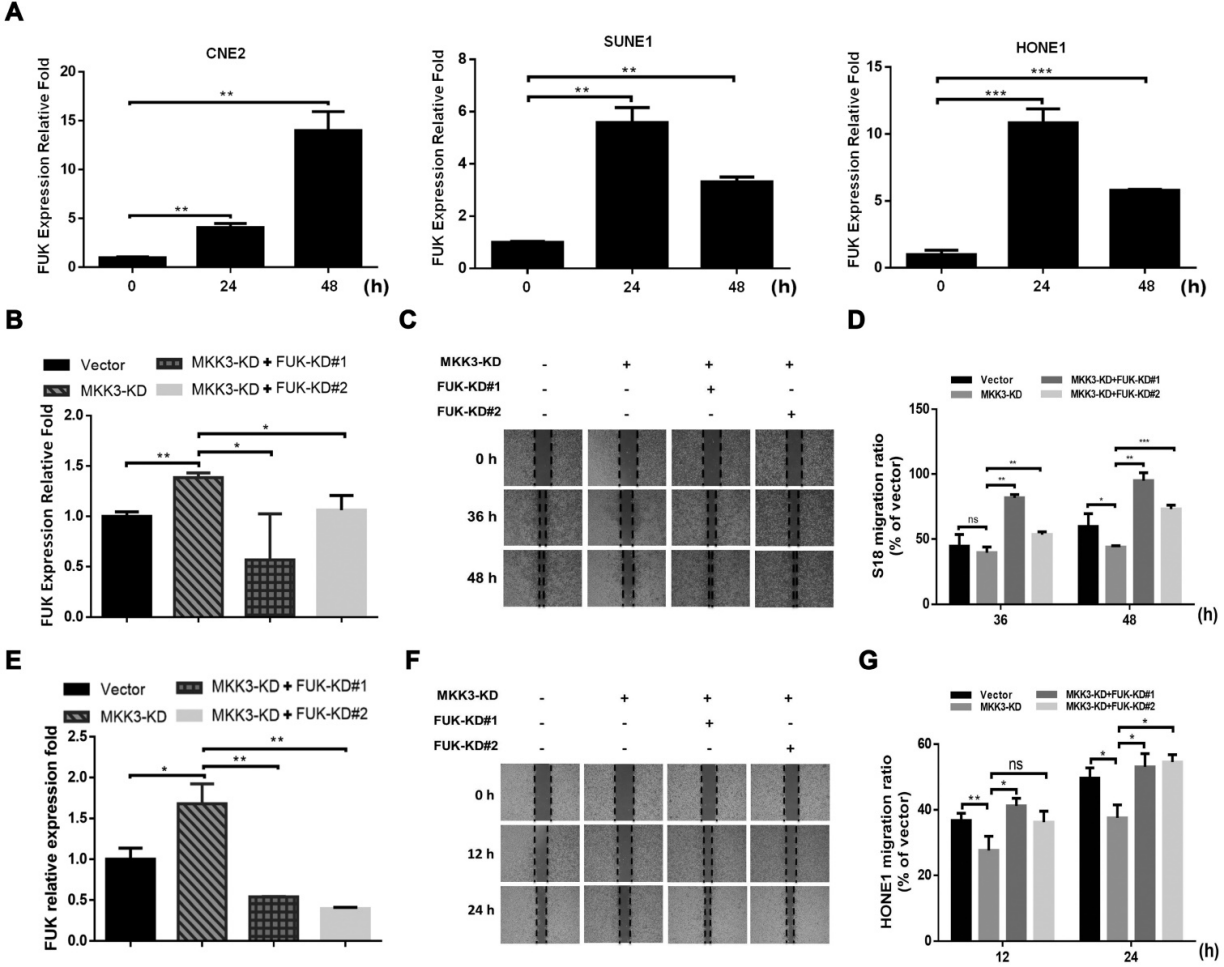
** MKK3-induced cell migration was inhibited by FUK upregulation.** (A) FUK mRNA expression levels were upregulated after capsaicin treatment in CNE2, SUNE1 and HONE1 cells. (B) MKK3 knockdown promoted FUK upregulation in S18 cells. (C) Knockdown of MKK3 suppressed cell migration. Dual knockdown of MKK3 and FUK reversed the cell migration capacity in S18. (D) Migration capacity was quantified from wound healing assay at 36 h and 48 h. (E) Knockdown of MKK3 promoted FUK up-regulation in HONE1. The knockdown efficiencies of MKK3 and FUK-specific shRNAs were checked by qPCR. (F) Knockdown of MKK3 suppressed cell migration in HONE1 cells, but dual knockdown of MKK3 and FUK restored cell migration. (G) Quantification of the wound healing assay at 12 h and 24 h. The data represent the means ± standard deviation. *p<0.05; **p<0.01; ***p<0.001.

Next, we screened metastasis-associated genes downstream of p38 after MKK3 knockdown in S18 and HONE1 cells. Here, FUK was significantly upregulated by both capsaicin treatment (75 μM; Figure 4A) and MKK3 knockdown (Figure 4B and E). Interestingly, the suppression of cell migration induced by MKK3 knockdown could be rescued by FUK knockdown in S18 (Figure 4C and D) and HONE1 cells (Figure 4F and G). Furthermore, S18 and HONE1 cells with dual MKK3 and FUK knockdown demonstrated greater cell migration capacities than both the MKK3 knockdown and control vector groups. However, cell viability of S18 and HONE1 was not affected by dual-knockdown of MKK3 and FUK (data not showed). These results suggest that the inhibitory effects of capsaicin on cell migration occurs via MKK3-p38 axis inhibition, in a FUK-mediated manner.

### Capsaicin inhibits tumor growth *in vivo*

**Figure 5:**
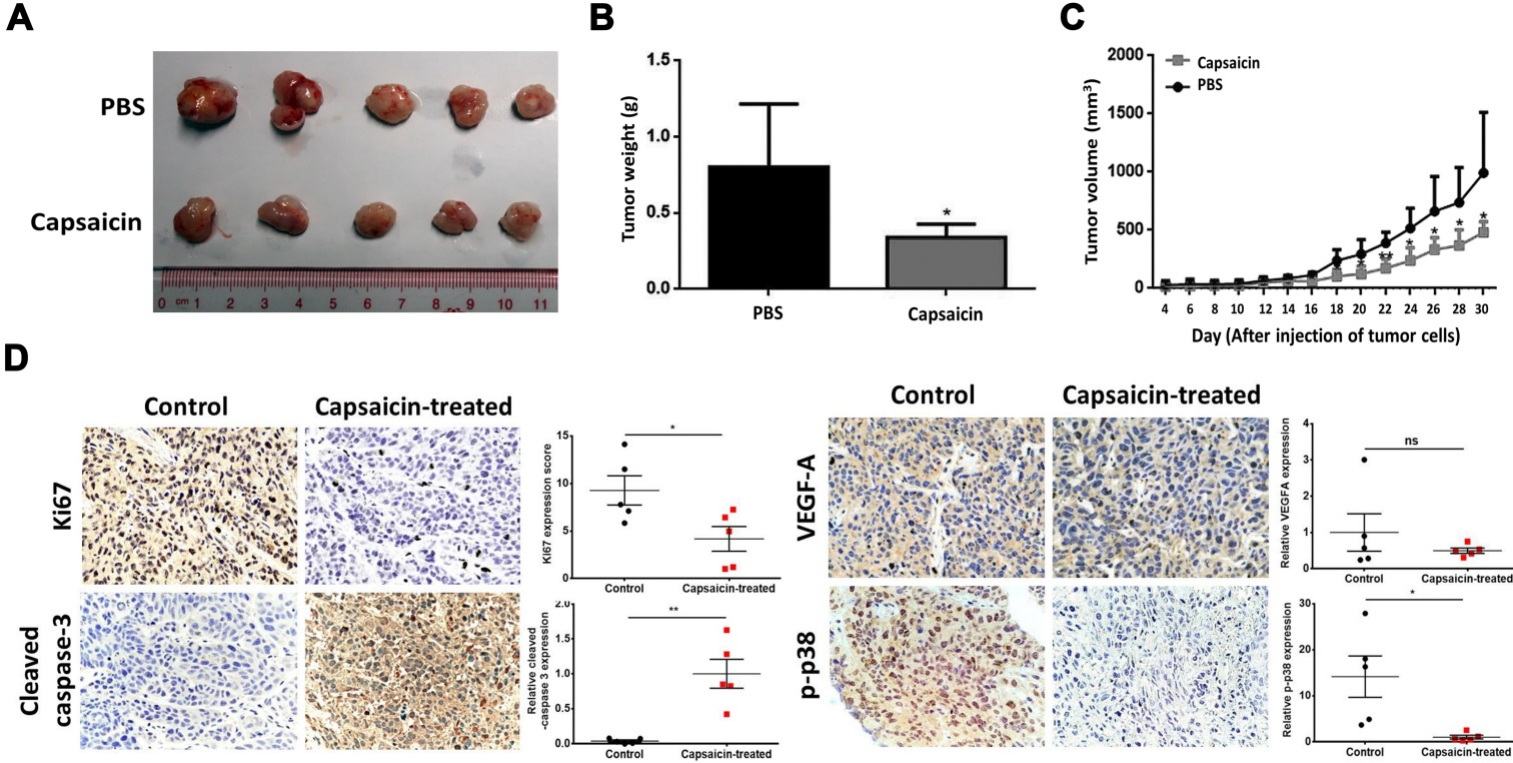
** Capsaicin exhibits anti-tumor effects on xenografts *in vivo*.** A total of 5 mg/kg capsaicin in 10% ethanol solution was administered orally every other day. The LD_50_ of capsaicin in male mice is 118.8 mg/kg. (A) The size of tumors in the control and capsaicin-treated groups. (B) The mean of tumor weights was measured at 30^th^-day after cancer cell injection. (C) The tumor volumes, measured every 2 days post-injection. (D) Immunohistochemical staining for Ki67, cleaved-caspase 3, VEGF-A and phospho-p38 expression to determine the status of cell proliferation, apoptosis, angiogenesis and the MKK3-p38 axis, respectively. The data represent the means ± standard deviation. *p<0.05; **p<0.01; ns, not significant.

Having shown the effects of capsaicin *in vitro*, we next wanted to determine the inhibitory effects *in vivo*. To do so, we subcutaneously injected SUNE1 cells (2×10^6^) into the flanks of nude mice and then exposed the mice to capsaicin treatment. Xenografts taken from mice in the capsaicin-treated group were significantly smaller than those taken from the PBS-treated group (Figure 5A and B). Indeed, we measured the tumor volume every 2 days after injection of the tumor cells, and found that it was significantly reduced in the capsaicin-treated group compared to the PBS-treated group (Figure 5C). However, there was no significant difference in the body weights between the two groups of mice (Figure S9), suggesting the dosage of capsaicin we used was systemic safety and no anti-obesity effect for mice.

We also analyzed the xenografts by immunohistochemical staining for Ki67, cleaved-caspase 3, VEGF-A and phospho-p38 to determine the status of cell proliferation, apoptosis, angiogenesis and the MKK3-p38 axis, respectively. Ki67 and phospho-p38 were downregulated in capsaicin-treated xenografts, whereas, cleaved-caspase 3 was upregulated; we saw no change in VEGF-A after capsaicin treatment. Taken together, these results support that capsaicin treatment via oral administration successfully inhibits tumor growth *in vivo*.

### Clinical relevance of phospho-MKK3

**Figure 6:**
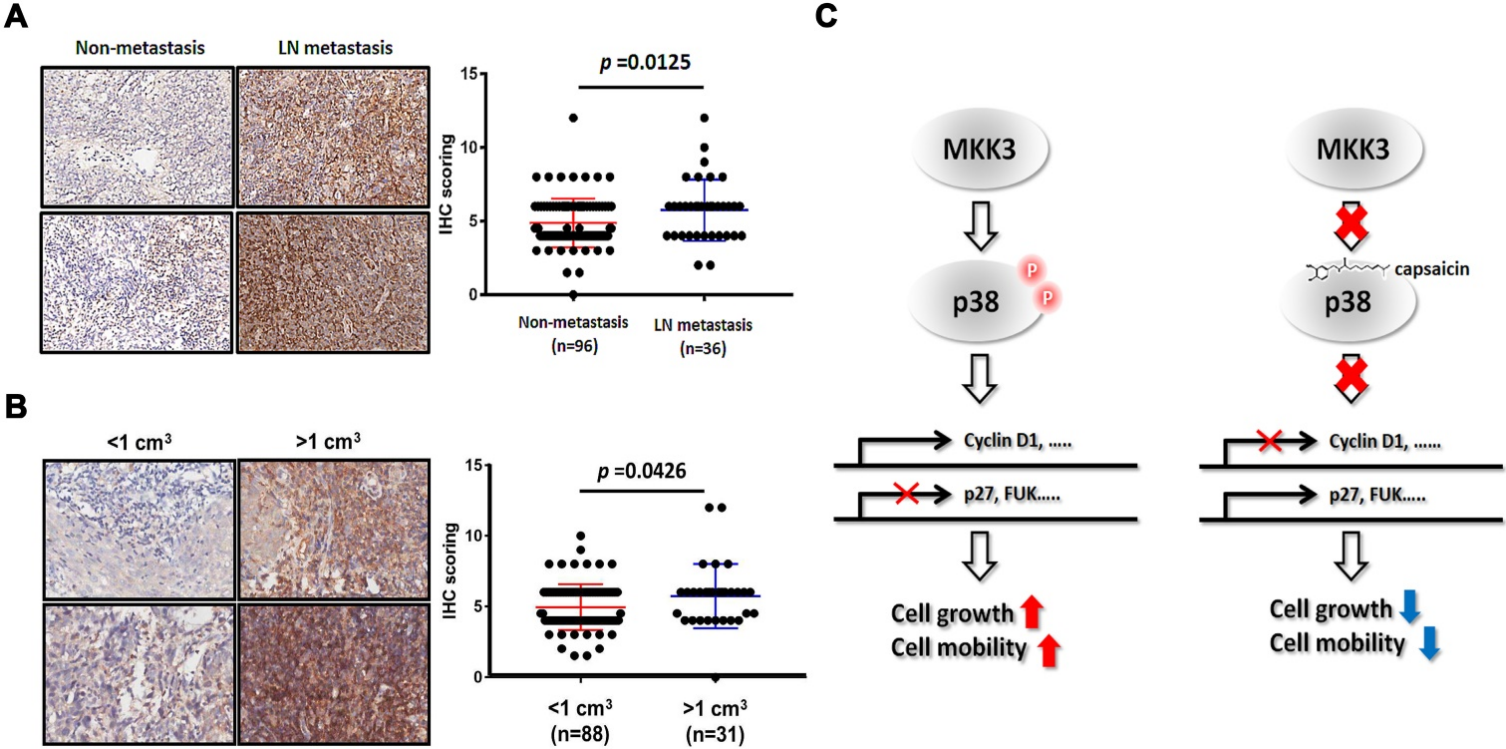
** Clinical impact of phospho-MKK3 in human NPC samples.** Immunohistochemical analysis of phospho-MKK3 expression in clinical NPC specimens, comparing patients with and without metastasis. (B) Immunohistochemical analysis of phospho-MKK3 expression in clinical NPC specimens, comparing patients with large and patients with small tumors. (C) A proposed molecular mechanism for the inhibitory effects of capsaicin on NPC progression. Left panel: p38 is phosphorylated by MKK3 and promotes the expression of cell cycle-associated genes. Cell-cycle inhibitors such as p27, or cell mobility inhibitors such as FUK, are suppressed in the absence of capsaicin. Right panel: Capsaicin binds directly to p38 and abolishes MKK3 signaling, resulting in reduced cyclin D1 expression and increased p27 and FUK expression. The data represent the means ± standard deviation. *p<0.05.

In our final analyses, we wanted to clarify the significance of the MKK3-related axis in the clinical context of NPC. We thus performed immunohistochemistry on 132 clinical NPC specimens, which included patients of all stages. We found that phospho-MKK3 protein expression was strongly, positively associated with clinical outcomes in patients with lymphoid node (LN) metastasis (Figure 6A, Table 1). We also found significantly increased levels of phospho-MKK3 in specimens from patients with LN metastasis (n = 36) compared with those without LN metastasis (n = 96, *p* = 0.0125). Significantly increased levels of phospho-MKK3 were also observed in specimens from patients with a large tumor volume (n = 31, >1 cm^3^) compared with those with a relatively small tumor volume (n = 88, <1 cm^3^; Figure 6B, Table 1; *p* = 0.0426). These results suggest that MKK3, and particularly the level of phospho-MKK3; is of substantial clinical relevance and is strongly associated with NPC progression, metastasis and growth. As shown in Figure 6C, targeting MKK3 with capsaicin might, therefore, represent a useful adjuvant to currently available treatments for NPC.

## Discussion

Capsaicin is frequently used as a herbal medicine, and its tumor-suppressive effects have been reported for a range of cancer types 42. However, capsaicin treatment has also been associated with gastrointestinal carcinogenesis 23, so its use as an anti-cancer agent is somewhat controversial. In addition, the mechanisms underlying its anti-cancer functions are yet to be fully elucidated. In the present study, we found that NPC cell growth was suppressed by capsaicin in a dose-dependent manner. Furthermore, expression of the cell-cycle marker cyclin D1 was downregulated by capsaicin treatment, while expression of the cell-cycle inhibitor p27^Kip1^ was upregulated. This finding suggests that capsaicin inhibits cell growth by inhibiting the G1-S phase transition, and by interfering with the interactions between cyclins and CDKs. These results are similar to those found previously in esophageal carcinoma 26, 43 and colon cancer 44.

Another well-known anti-tumor effect of capsaicin is its capacity to induce apoptosis 45; this effect has been demonstrated in pancreatic 46, colon 47, prostate 48, liver 49 and esophageal cancer 26. Our findings mean that we can now add NPC to this list. Furthermore, we found that capsaicin suppresses cell migration and invasion of cultured NPC cells. Previous studies have described similar effects of capsaicin in the context of colon cancer 50, bladder cancer 51 and melanoma 52.

The MKK3-p38 axis is well known for its involvement in cancer progression. Interestingly, the p38-MAPK pathway is downregulated following capsaicin treatment in human invasive fibrosarcoma 31. We thus decided to investigate the effects of capsaicin on this pathway in the context of NPC. The results of immunoprecipitation assays revealed that MKK3 overexpression, but not MKK6, increased p38 phosphorylation levels. Furthermore, p38 phosphorylation decreased following capsaicin treatment in both NPC and HEK293T-MKK3 overexpressing cells. Thus, the interactions between MKK3 and p38 *in vitro* seem to be directly interrupted by capsaicin. Indeed, pull-down assays and computer modeling approaches revealed that p38 is a novel target for capsaicin. Functional assays further demonstrated that MKK3-driven cancer progression is critical in NPC and that MKK3-induced cell migration and invasion can be effectively inhibited by capsaicin treatment. The efficacy of this treatment was similar to that of treatment with p38-specific inhibitors.

Further mechanistic analyses showed that FUK, a downstream gene of the p38-ATF2 axis, is suppressed by p38 phosphorylation. According to previous studies, FUK is a negative regulator of cell motility, cell growth and spheroid formation in melanoma 39. In the context of NPC, we found that FUK is upregulated by both capsaicin treatment and MKK3 knockdown in NPC cells, and that the suppression of NPC cell motility is due to FUK upregulation. Furthermore, we could restore NPC cell motility through dual MKK3 and FUK knockdown. Taken together, these results suggest that FUK has a central role in mediating the motility-inhibiting effects of capsaicin.

In the clinical context, we found that phosphorylated MKK3 expression positively correlates with tumor growth and lymphoid node metastasis in clinical NPC specimens. This result seems to concur with those of other studies in the context of melanoma 36, breast cancer 33 and hepatocellular carcinoma 32. It thus seems that the MKK3-associated signaling pathway is activated and vital to NPC tumorigenesis. The deceleration of cell growth and migration by MKK3 knockdown in NPC cell lines was also consistent with our clinical evidence. In stable MKK3-knockdown cells, altered expression of various EMT markers was associated with increased cell migration and invasion capacities *in vitro*.

Overall, the results of our study support that p38 is a novel target for capsaicin. Capsaicin treatment inhibits signal transduction from MKK3 to p38, and through blocking this interaction can inhibit NPC progression. MKK3 signaling pathway activity seems to be markedly associated with primary tumor growth and NPC cell growth *in vitro*. Our observations that tumor growth *in vivo* is suppressed by capsaicin treatment are consistent with our *in vitro* evidence.

To the best of our knowledge, our study is the first to report that MKK3 is a key regulator of NPC development and progression. We propose that the levels of phosphorylated MKK3 might represent a novel therapeutic biomarker for NPC, and that phospho-MKK3 expression status might serve to identify patients in need of intensive treatment. Our findings also support the potential for capsaicin and MKK3-p38 pathway targeting as a novel NPC treatment modality. Because capsaicin interacts with other anti-cancer agents in a synergistic manner 53, 54, we speculate that it might be used as an adjuvant alongside chemotherapeutic agents to increase their overall efficacy. Further studies are now warranted to fully elucidate the underlying inhibitory mechanisms of capsaicin in NPC.

## Materials and Methods

### Reagents, enzymes and antibodies

Capsaicin (HY-10448) and Capsazepine (CPZ) were purchased from MedChemExpress LLC (Monmouth Junction, NJ, USA). Chemical reagents and reaction buffers were obtained from Sigma-Aldrich; Merck KGaA (Darmstadt, Germany). The p38 inhibitor LY2228820 was obtained from Target Molecule Corp. (Wellesley Hills, MA, USA). DNA polymerase and DNA ligase were obtained from Qiagen (Valencia, CA, USA) and Takara Bio, Inc. (Kusatsu, Shiga, Japan), respectively. Fetal bovine serum (FBS) was obtained from PAN-Seratech GmbH (Aidenbach, Germany). Cell culture media and other reagents were from Life Technologies; Thermo Fisher Scientific, Inc. (Waltham, MA, USA).

The restriction enzymes EcoRI (R3101S), Bam HI (R3136S), XbaI (R0145S) and XhoI (R0146S) were purchased from New England BioLabs (Ipswich, MA, USA). Antibodies list was provided in Supplementary Information.

### Cell culture and stable pool selection

HEK293T cells (obtained from American Type Culture Collection, Virginia, USA) were cultured in Dulbecco's modified essential medium (DMEM) with 10% FBS and 100 U/ml penicillin-streptomycin. The human NPC cell lines CNE2, SUNE1, S18 and HONE1 (Sun Yat-Sen University Cancer Center, Guangzhou, China) were cultured in RPMI1640 containing 10% FBS and 100 U/ml penicillin-streptomycin. All cell lines, including stable pool cell lines, were propagated in an incubator set at 37˚C with 5% CO_2_ and were authenticated by short tandem repeat profiling (Guangdong Huaxi Forensic Physical Evidence Judicial Appraisal Institute, Shenzhen, China). The cell lines were passaged <10 times, <6 months after initial revival from frozen stocks, and tested negative for mycoplasma contamination (Qiagen). MKK3 over-expression and knockdown stable pools were selected using 10 μg/ml puromycin (Selleck Chemicals, Houston, TX, USA) in RPMI1640 complete medium for 1 week, and MKK3 expression was assessed by qPCR and western blot analysis (as described below). Functional assays were performed after the expression levels were confirmed.

### Plasmid construction

Full-length human MKK3 and MKK6 cDNA were amplified from a human mRNA pool by RT-PCR using SuperScript II RNase H Reverse Transcriptase (Life Technologies; Thermo Fisher Scientific, Inc.). MKK3 and MKK6 cDNAs were cloned into the *EcoRI/NotI* and the *XbaI/NheI* sites of pCDH-puro vectors, respectively. The sequences were confirmed by Sanger sequencing (Genewiz, Guangzhou, China). The sequences of the cloning primers are provided in the Supplementary Information.

### MKK3 and FUK knockdown

MKK3- and FUK-knockdown cells were generated through lentiviral-mediated delivery of either MKK3 or FUK small hairpin (sh)RNAs. The shRNA oligos were synthesized by Life Technologies (Thermo Fisher Scientific, Inc.) and cloned into a pLKO.1 expression construct (using pLKO.1-scramble shRNA as a control). The shRNA sequences used are provided in the Supplementary Information. The resultant pLKO.1-shRNA plasmids were co-transfected into HEK293T cells with the pCMV-VSV-G packaging plasmids and pCMV-delta-8.2 envelope plasmids to produce the shRNA lentivirus, as described below. After 48 h, the supernatant fractions from the cell cultures were collected and filtered through a 0.45 μm filter. The cells were then infected with the viral supernatant fractions and supplemented with polybrene. The culture medium was replaced with fresh growth medium 16 h post-infection, with 2 μg/ml puromycin for 48 h selection. The cells were cultured in this medium until the control cells died. The knockdown efficiency was then evaluated by qPCR and western blotting.

### Transient transfection

A total of 5×10^5^ HEK293T cells were inoculated in a 100-mm dish and allowed to reach 70% confluence. Then, Lipofectamine 3000™ and the target plasmids were added and transfected according to the manufacturer's protocol (Life Technologies; Thermo Fisher Scientific, Inc.), and configured in opti-MEM for 10 min at room temperature. The transfection mixture was incubated with the cells for 48 h, and gene and protein expression were detected by qPCR or western blotting, respectively.

### Total RNA isolation

A total of 5×10^5^ cells were lysed in 1 mL TRIzol reagent, and 200 μL chloroform was applied to separate the RNA at the aqueous phase. To precipitate the RNA, 500 μL isopropanol was added, and the RNA was centrifuged at 14,000 rpm for 15 min. Finally, the RNA was dissolved in DEPC-MQ H_2_O.

### RT-PCR

Isolated RNA (3 μg) was added to a sterile RNase-free microcentrifuge tube, and supplemented with 1 μg Oligo (dT) to a total volume of 5 μL. The tubes were heated to 70°C for 5 min to destroy the secondary RNA structure and then returned to room temperature. After a brief centrifugation, the following components were added to the annealed primers: M-MLV RT 5× reaction buffer (5 μL), dNTP (10mM, 1 μL), M-MLV RT (1 μL, 200 units), and nuclease-free water to a final volume of 20 μL. The tubes were gently mixed and were then incubated at 42°C for 1 h and then at 95°C for 5 min. The RT-PCR products were diluted 10-fold before further use. For qPCR, templates were collected from the cDNA extracted from the cell lines, and 1 μL cDNA template was used for PCR. The PCR mixture contained 10× reaction buffer (2 μL), 10 mM dNTPs (1 μL), 10 mM oligonucleotide primers (0.5 μL each), and Taq polymerase (0.2 μL). Then, sterile MQ-H_2_O was added to a final volume of 20 μL.

### Real time qPCR

cDNA obtained via reverse transcription was diluted 10-fold, and prepared according to the SYBR Green Reagent specification (Vazyme Biotech co., Ltd., Nanjing, China). Each qPCR reaction was repeated three times, and the expression of the target genes was normalized to the internal reference GAPDH. The thermal cycling was as follows: 95°C for 10 min; 40 cycles of 95°C for 15 s and 60°C for 30 s. The mRNA expression of the target genes was analyzed using the 2^-ΔΔC^T method. The primers used for qPCR are provided in the Supplementary Information.

### Protein extraction, immunoprecipitation and western blot analysis

For immunoprecipitation, HEK293T cells transfected with Flag-tagged MKK3 or HA-tagged MKK6 were cultured in a 100-mm dish, and the cells were collected with IP lysis buffer once they reached 80% confluence. After clarification, the supernatant fractions were used for immunoprecipitation with antibodies targeting HA or Flag, and were centrifuged at 5 rpm at 4°C overnight. Western blot analysis was performed after the immunoprecipitation. Total protein was extracted from the MKK3-overexpressing and Knockdown stable pools in sample buffer containing 1% SDS. The protein concentration was determined using a Bio-Rad DC Protein Assay (Bio-Rad Laboratories, Inc., Hercules, CA, USA), according to the manufacturer's protocol. All protein samples were separated by electrophoresis using a Ready Gel System (Bio-Rad Laboratories, Inc.) and then transferred to polyvinylidene fluoride membranes. The membranes were labeled with the corresponding antibodies, and protein expression was detected using Pierce ECL western blotting substrate (Thermo Fisher Scientific, Inc.).

### Protein pulldown assay

Capsaicin was modified by biotin-labeling and conjugated with streptavidin magnetic beads (cat. no. HY-K0208, MedChemExpress LLC, Monmouth Junction, NJ, USA). SUNE1 cell lysates were prepared using RIPA lysis buffer containing protease inhibitors, and were incubated with capsaicin-conjugated beads at 4°C overnight. Washing buffer was applied to remove non-specific protein binding, and the captured proteins were detected by western blotting.

### Computer modeling analysis

Computational docking was performed using Audodock Vina 55. The crystal structures of MKK6 (3VN9), unphosphorylated-p38 (5LAR) and phosphorylated-p38 (3PY3) were obtained from the Protein Data Bank (PDB) 56. Protein preparations were performed using AudoDock MGL Tools (version 1.5.6). The search space for docking was large enough to include the default pocket of each target protein and for the ligand to rotate in. Potential binding poses were selected based on their binding affinity energy.

### Cell proliferation and clonogenic assays

A Cell Counting Kit-8 (CCK-8) assay was used to assess cell proliferation. Briefly, cells were seeded in a 96-well plate at a concentration of 1-2×10^3^ cells per well, and were incubated in a final volume of 100 μL culture medium per well. Cell viability was evaluated at 0, 24, 48 or 72 h after cell attachment. CCK-8 reagent (10 μL; Dalian Meliun Biotech Co., Ltd., Dalian, China) was added to each well for 2 h before the absorbance of the resultant formazan product was measured at 450 nm.

For the clonogenic assays, the cells were seeded in a 6-well plate at a concentration of 500 cells/well. The cells were incubated in a final volume of 4 mL culture medium per well for 8-10 days, following which the plates were washed in PBS and the cells were fixed with 100% methanol at 4°C for 10 min. Then, the cells were stained with 0.05% crystal violet for 10 min at room temperature before washing out with ddH_2_O twice.

### Wound healing assays

Cells were seeded into 6-well plates and cultured until 90% confluence was reached. Following this, they were starved overnight in serum-free medium, and the cell monolayer was scratched using a sterile 200 μL tip. The plates were then washed 2-3 times with PBS to remove all floating cells. The RPMI1640 medium (containing 2% FBS) was changed after scratching. Wound healing was monitored under an inverted microscope (Olympus IX73; Olympus Corporation, Tokyo, Japan), and images of the migrating cells were captured at 0 and 48 h.

### Cell migration and invasion assays

Cell migration and invasion assays were performed using Transwell chambers (8-mm pores, Corning, Inc., Corning, NY, USA) pre-coated without (migration assay) or with (invasion assay) 70 μL Matrigel (BD Biosciences, Franklin Lakes, NJ, USA; Matrigel:serum-free medium = 1:5). Either 5×10^4^ or 1×10^5^ cells suspended in 200 μL serum-free medium were seeded in the upper chambers, and 600 μL medium supplemented with 10% FBS was placed in the lower chambers. After incubation for 12-36 h, the cells on the upper surface of the membrane filter were fixed with methanol and stained with 0.05% crystal violet. Then, the non-migrating cells were removed with a cotton swab. Images of the migrating cells in five random fields were captured using an Olympus IX73 inverted microscope (Olympus Corporation).

### Apoptosis assays

Cells were seeded into 6-well plates (3×10^5^ cells/well) in complete RPMI1640. This medium was replaced by serum-free medium after 4 h, and the cells were starved overnight. The following day, the cells were treated with varying doses (0~75 μM) of capsaicin at different times (0~48 h). Following treatment, the number of cells remaining were counted. Then, the cell concentration of each group was adjusted to be equal, and the cells were washed with PBS. The cells were then suspended in 500 μL binding solution containing 5 μL annexin-V FITC and 10 μL PI dye, and incubated in the dark for 5 min. Cell apoptosis was measured via flow cytometry using the FACS Calibur platform (BD Biosciences).

### Xenografts in nude mice

All animal research procedures were performed according to the protocols of the Animal Care and Use Ethics Committee of Shenzhen University Health Science Center and all animals were treated in strict accordance with protocols approved by the Institutional Animal Use Committee of the Health Science Center, Shenzhen University. Male BALB/c nu/nu mice (Charles River, Beijing, China, ~4-6 weeks old; n=10) were subcutaneously injected with 2×10^6^ SUNE1 cells on the right side of the back. The mice were then randomly divided into control (n=5) and experimental (n=5) groups. The day after injection, PBS buffer (containing 0.1% ethanol; control group) or capsaicin (5 mg/kg; experimental group) was administered orally 57, three times per week. The tumor volume was measured three times per week using the following formula: volume = length × width^2^ / 2. The mice were sacrificed after 4 weeks of tumor growth, or when the tumors reached >1 cm in diameter at their widest point. The tumor xenografts were subsequently harvested.

### Tissue microarray (TMA) and immunohistochemistry (IHC)

TMA sections containing tissues from 132 human patients with NPC (HNasN132Su01) were obtained from Shanghai Outdo Biotech Co. Ltd. (Shanghai, China). IHC was then performed to examine the phospho-MKK3 expression profile. The TMA sections were labelled with an phospho-MKK3 (1:50) antibody. phospho-MKK3 staining was scored by two independent pathologists blinded to the patient characteristics. Staining intensity was classified as 0 (negative), 1 (weak), 2 (moderate) or 3 (strong); while staining extent was based on the percentage of 200 cells that were phospho-MKK3^+^: 0 (5%), 1 (5-25%), 2 (26-50%), 3 (51-75%) or 4 (75%-100%). According to these scores, the results of IHC were classified as 0-1, negative (-); 2-4, weakly positive (+); 5-8, moderately positive (++) and 9-12, strongly positive (+++).

IHC was also performed to detect the expression profile of Ki67, cleaved-caspase 3, VEGF-A and phospho-p38 in xenograft tissues. The xenograft tissues were stained with antibodies against Ki67 (1:100), cleaved-caspase 3 (1:50), VEGF-A (1:50) and phospho-p38 (1:50). Then, the IHC positive cell percentage was analyzed using Image-Pro Plus 6.0 software in at least three 200-fold fields of vision that were randomly selected for each slice in each group. The positive rate (%) was calculated using the following formula: positive cell number/ total cell number * 100%. The cumulative optical density value (IOD) and the positive pixel area (AREA) was also analyzed using Image-Pro Plus 6.0 software in at least three 200-fold fields of vision that were randomly selected for each slice in each group. The average optical density (AOD) was calculated using the following formula: IOD / AREA.

### Statistical analyses

All *in vitro* experiments were performed at least three times. The results of each experiment are presented as the means ± standard deviation. Data analyses were performed in Microsoft Excel 2010 Professional Plus (Version 14.0.7237.5000) and Prism 6 (Version 6.01). Two-tailed, paired or unpaired Student's t-tests were used to compare the differences between two groups with similar variance. For two groups with standard deviations ~1 and average differences >3, the effects can be detected with a sample size of 3. For all tests, a *p*<0.05 was considered to indicate a statistically significant difference.

## Supplementary Material

Supplementary figures and tables.

## Figures and Tables

**Table 1:** Clinicopathological characteristics of patients with NPC

Characteristic	No. of patients with characteristic		*p* value
Age (years)	>50 (n=78)	<50 (n=54)	0.7393
Sex	male (n=101)	female (n=31)	0.0192*
Cancer stage	I+II (n=72)	III+IV (n=60)	0.3096
Tumor size (mm^3^)	>1 (n=88)	<1 (n=31)	0.0426*
Survival time (months)	>60 (n=10)	<60 (n=122)	0.0994
Recurrence	recurrence (n=71)	non-recurrence (n=61)	0.173
LN metastasis	metastasis (n=96)	non-metastasis (n=36)	0.0125*

*p<0.05.

## Abbreviations

MKK3: Dual specificity mitogen-activated protein kinase kinase 3
p38: Mitogen-Activated Protein Kinase P38
NPC: Nasopharyngeal carcinoma
FUK: Fucokinase
EBV: Epstein-Barr virus
SNPs: Single nucleotide polymorphisms
TNM: Tumor-node-metastasis
PI3K: Phosphatidylinositol-4,5-Bisphosphate 3-Kinase
Akt: AKT Serine/Threonine Kinase
MKK6: Dual specificity mitogen-activated protein kinase kinase 6
ATF2: Activating Transcription Factor 2
p27: Cyclin Dependent Kinase Inhibitor 1B
p21: Cyclin Dependent Kinase Inhibitor 1A
qPCR: Quantitative polymerase chain reaction
CCK-8: Cell counting kit-8
ZO-1: Zona Occludens Protein 1
ZEB1: Zinc finger E-box binding homeobox 1
FBS: Fetal bovine serum
PBS: Phosphate buffered saline
DMEM: Dulbecco's modified essential medium
RPMI1640: Roswell Park Memorial Institute 1640
M-MLV RT: Moloney murine leukemia virus reverse transcriptase
dNTP: deoxy-ribonucleoside triphosphate
GAPDH: Glyceraldehyde-3-phosphate dehydrogenase
HA: Hemagglutinin
Rpm: Revolution(s) per minute
SDS: Sodium dodecyl sulfate
ECL: Enhanced chemical luminescence
RIPA: Radioimmunoprecipitation assay buffer
PDB: Protein Data Bank
ddH2O: Double-distilled water
PI: Propidium iodide
TMA: Tissue microarray
IHC: Immunohistochemistry
