# Supplementary material for: Therapeutic potential of targeting MKK3-p38 axis with Capsaicin for Nasopharyngeal Carcinoma
Source: Theranostics. 2020 Jun 24;10(17):7906–20. doi: 10.7150/thno.45191 (PMC7359099; doi:10.7150/thno.45191)
Supplement: Supplementary file 1 — Supplementary figures and tables. [file thnov10p7906s1.pdf]

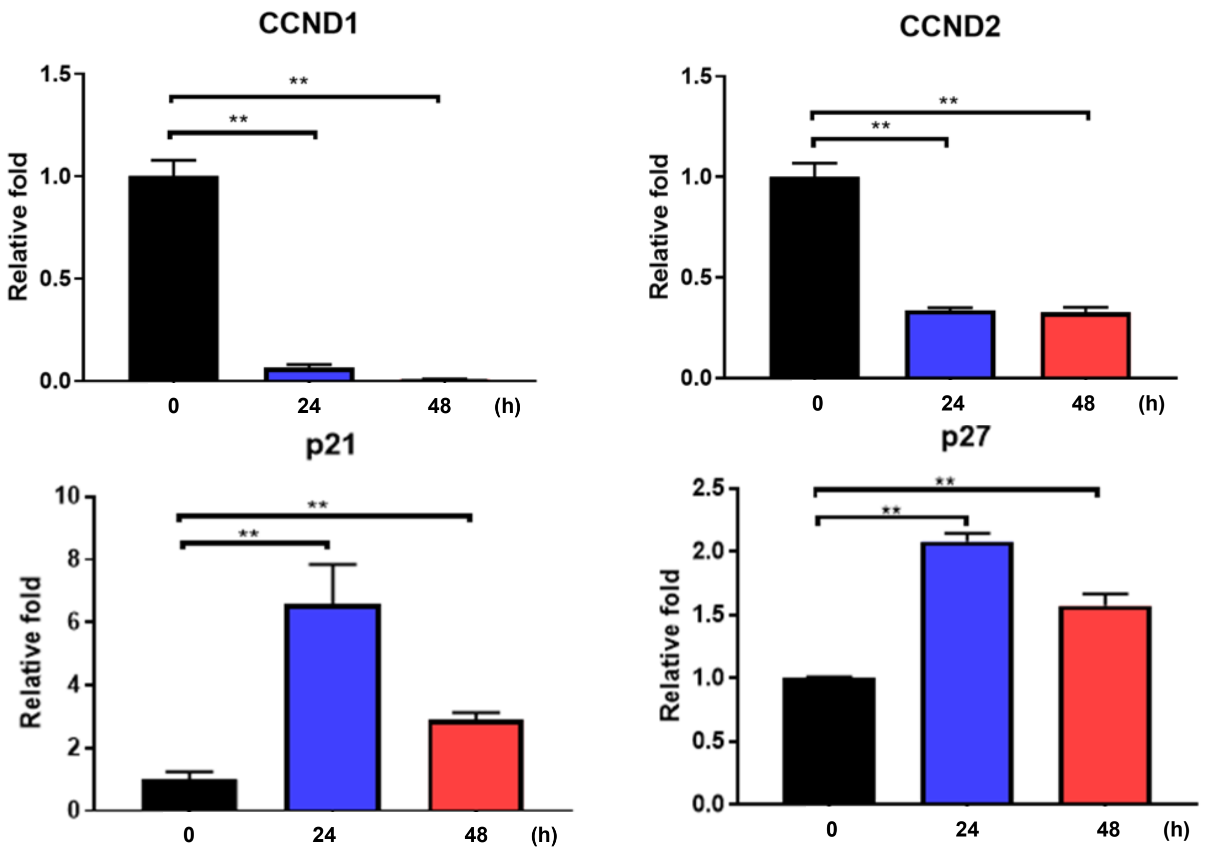

Supplementary Figure S1. Expression of proliferation-associated genes downstream of p38 in NPC cell lines after capsaicin treatment.

CCND1 and CCND2 expression was significantly downregulated following capsaicin treatment, while p21 and p27 were upregulated. The data represent the means ± standard deviation. $^{**}P<0.01$.

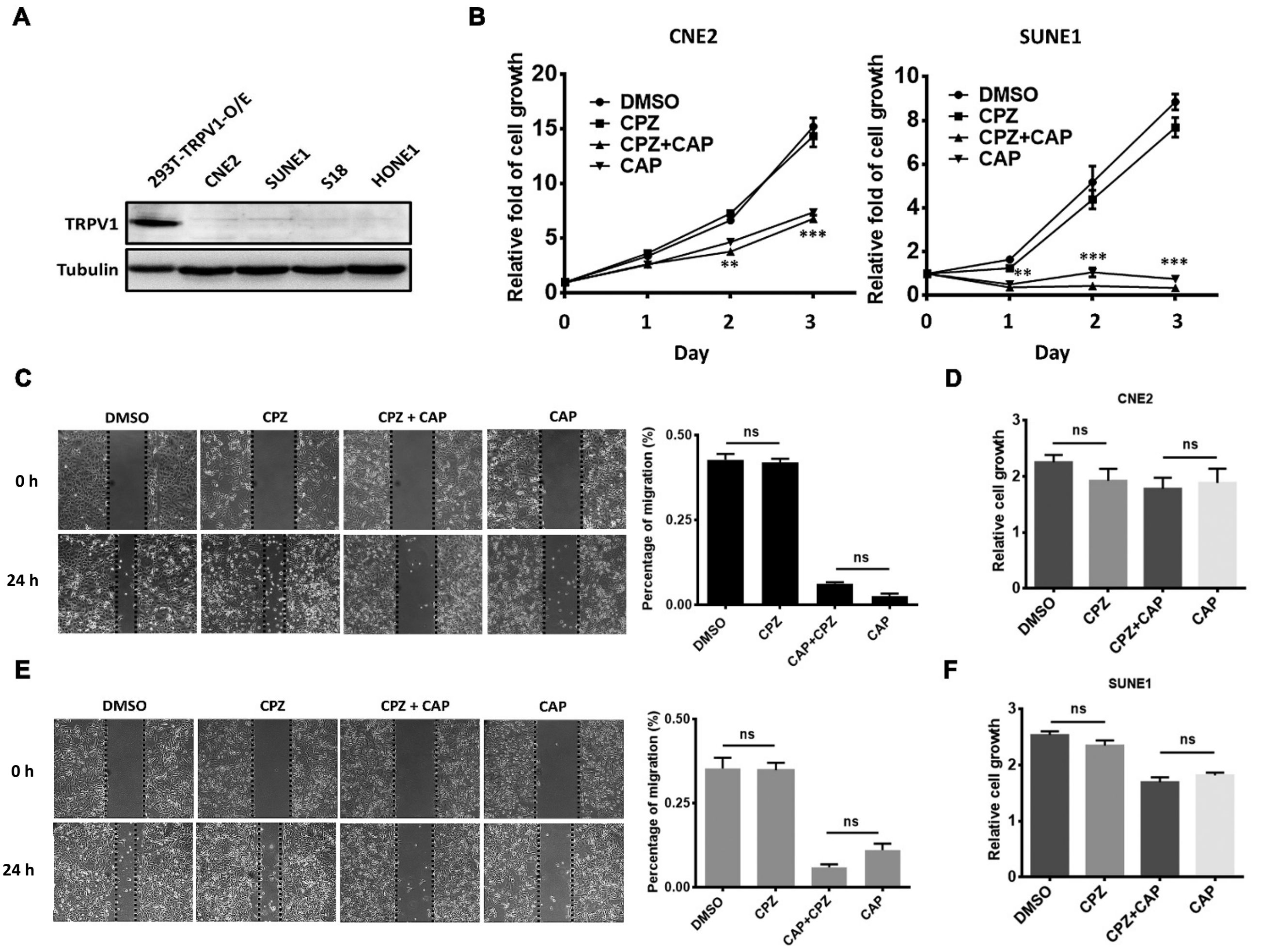

Supplementary Figure S2. Capsaicin exerts anticancer effects in a TRPV1-independent manner in NPC cells.

(A) TRPV1 expression was detected by western blotting in NPC cell lines. (B) NPC cells were pretreated with CPZ (30 μM) for 2 h prior to capsaicin (50 μM) treatment for 24, 48, or 72 h (10% FBS); cell growth was detected by CCK8 assay. (C) The cell migration capacity of CNE2 was measured after CPZ (5 μM) pretreatment, before capsaicin (75 μM) treatment for 24 h (2% FBS). (D) The cell growth of CNE2 was detected under the same conditions as described for (C). (E) The cell migration capacities of SUNE1 was measured after CPZ (5 μM) pretreatment, before capsaicin (75 μM) treatment for 24 h (2% FBS). (F) The cell growth of SUNE1 was detected under the same conditions as described for (E). The data represent the means ± standard deviation. *, $p<0.05$; **, $p<0.01$; ***, $p<0.001$; ns, not significant.

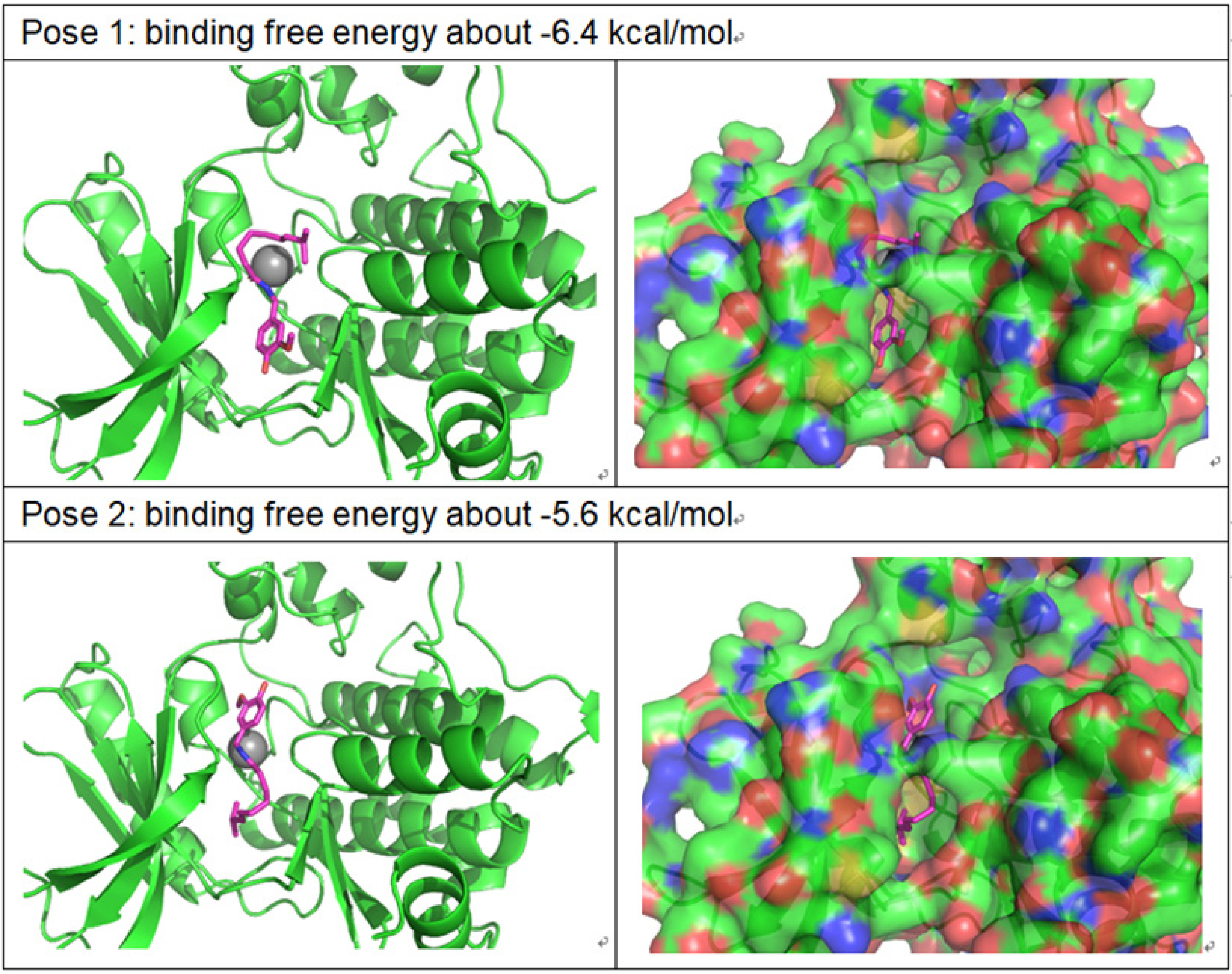

Supplementary Figure S3. Computer modelling of capsaicin binding with MKK6.

In pose 1, the kinetic free energy of capsaicin binding with MKK6 was -6.4 kcal/mol (top). In pose 2, the kinetic free energy of capsaicin binding with MKK6 was -5.6 kcal/mol (bottom).

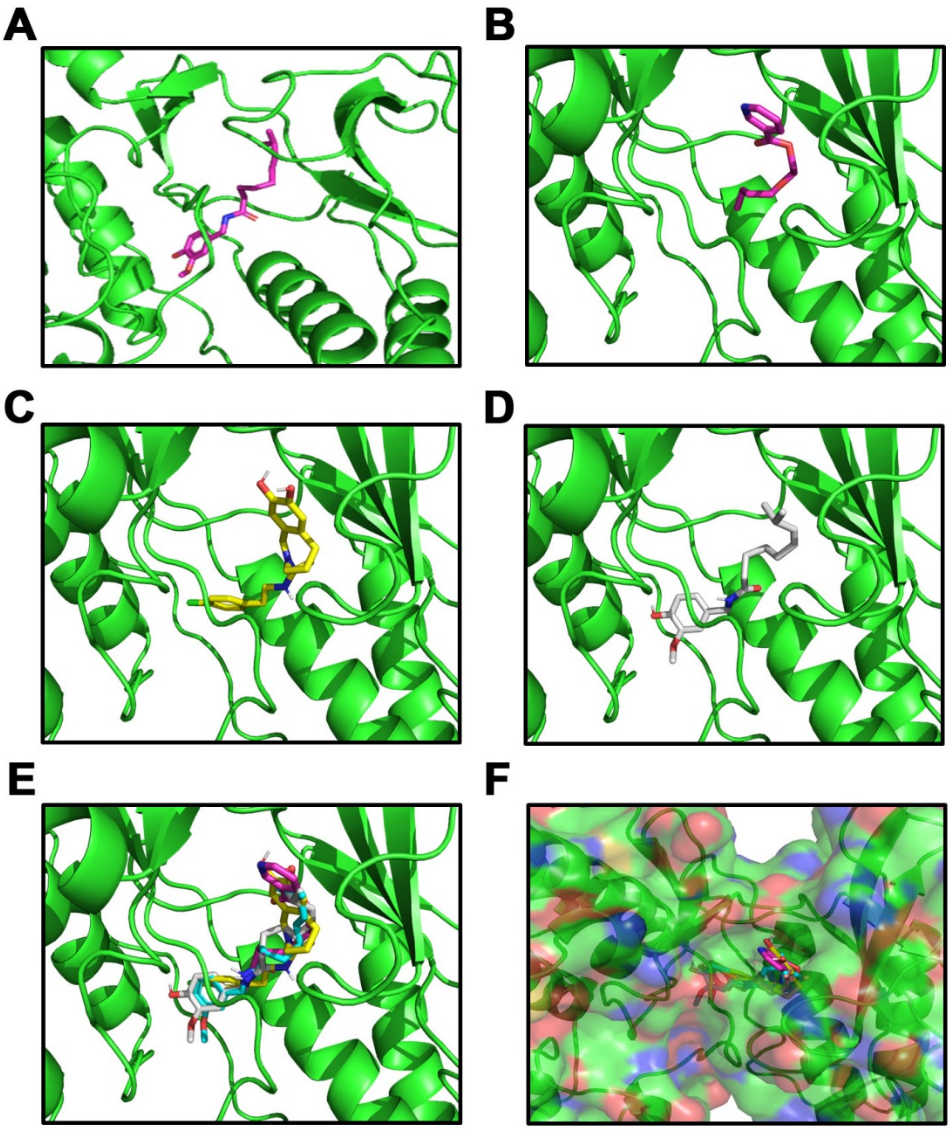

Supplementary Figure S4. Computer simulation of p38 binding with capsaicin analogues.

Capsaicin and other three capsaicin analogues were docked into the p38 pocket. (A) Capsaicin; (B) Nicoboxil; (C) Capsazepine; (D) Zucapsaicin; (E) Superposed ligands; (F) Superposed ligands in the p38 pocket (protein surface shown). The binding free energies of Capsaicin, Nicoboxil, Capsazepine and Zucapsaicin with p38 were -12.3, -6.4, -10.5 and -8.9 kcal/mol, respectively.

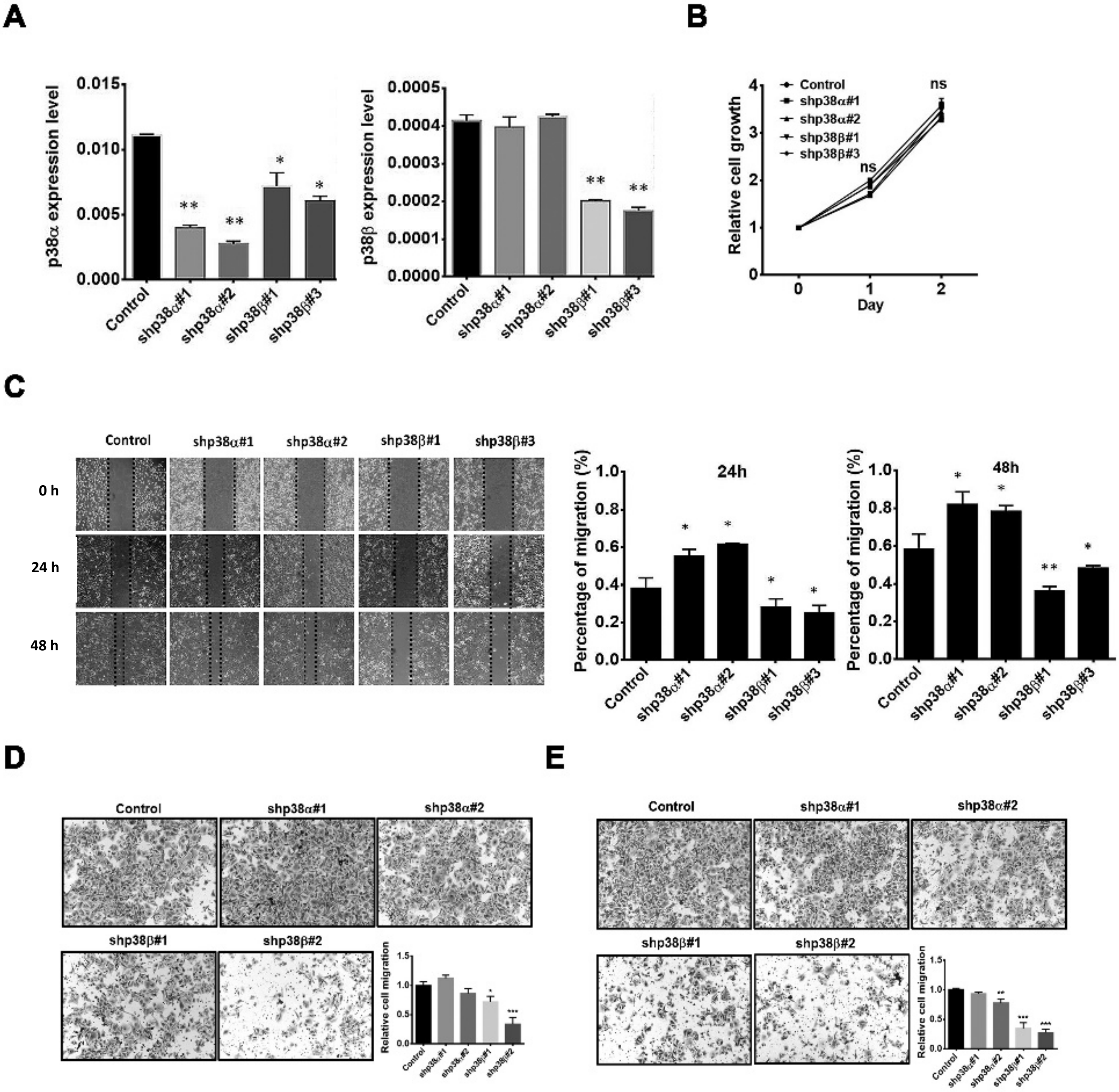

A
p38α expression level
0.015
0.010
0.005
0.000
Control
shp38α#1
shp38α#2
shp38β#1
shp38β#3
p38β expression level
0.0005
0.0004
0.0003
0.0002
0.0001
0.0000
B
Control
shp38α#1
shp38α#2
shp38β#1
shp38β#3
ns
Relative cell growth
Day
C
0 h
24 h
48 h
24h
48h
Percentage of migration (%)
D
Control
shp38α#1
shp38α#2
shp38β#1
shp38β#2
Relative cell migration
E
Control
shp38α#1
shp38α#2
shp38β#1
shp38β#2
Relative cell migration

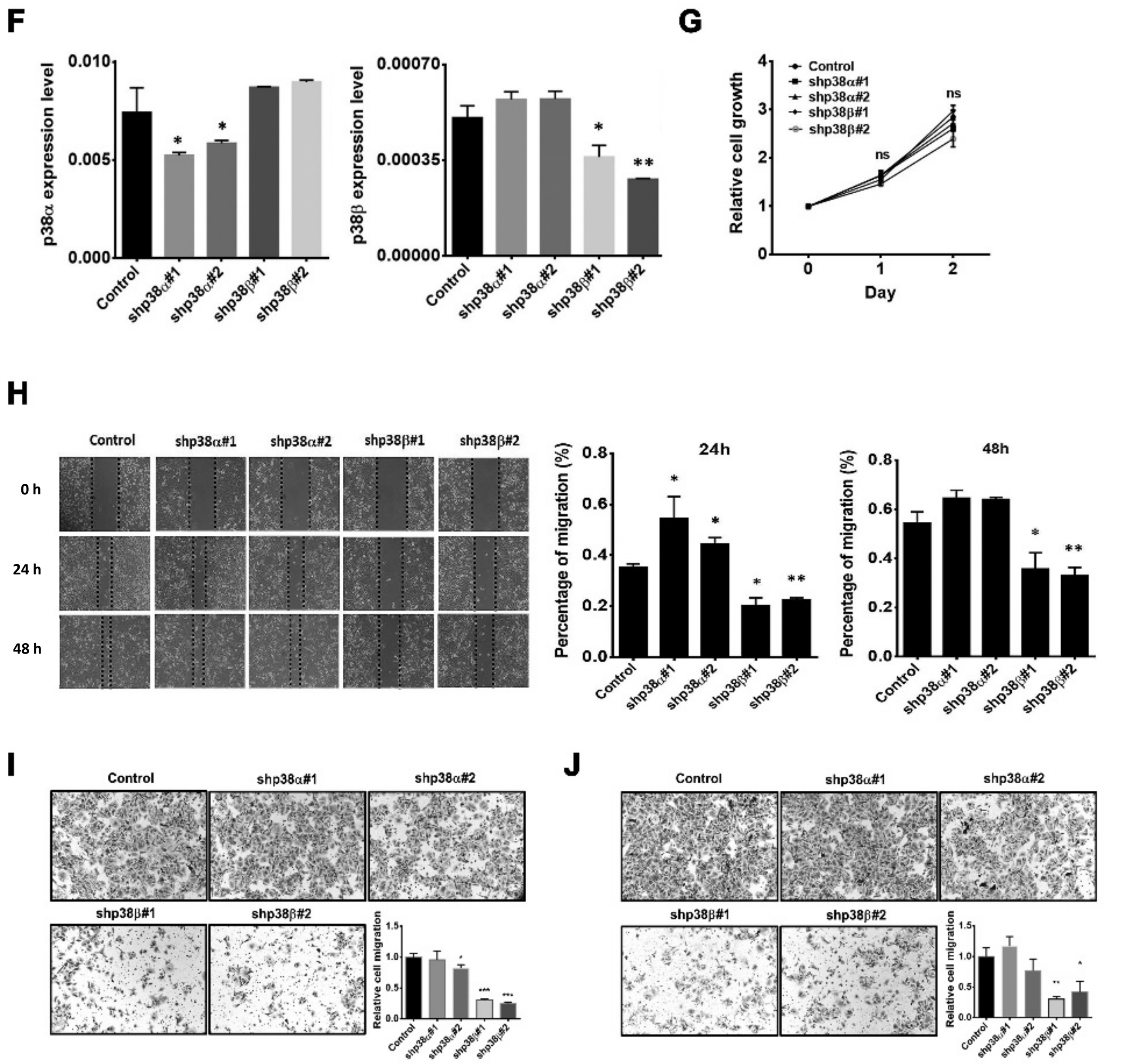

Supplementary Figure S5. Cell migration capacities of CNE2 and SUNE1 MKK3-over-expressing stable cells after p38α and p38β knockdown.

(A) The p38α and p38β expression levels in CNE2 MKK3-over-expressing stable pools after p38α- and p38β-knockdown were detected by qPCR. (B) Cell growth was determined by CCK-8 assay in 2% FBS. (C) A wound healing assay was performed in 2% FBS. The cell migration capacity of CNE2 MKK3-over-expressing stable cells after p38α and p38β knockdown was monitored at 24 h and 48 h. (D) Cell migration and (E) invasion capacities were performed in 2% FBS and measured at 21 h. (F) The p38α and p38β expression levels in SUNE1 MKK3-over-expressing stable pools after p38α- and p38β-knockdown were detected by qPCR. (G) Cell growth was assessed in 2% FBS. (H) A wound healing assay was performed in 2% FBS. The cell migration capacity of SUNE1 MKK3-over-expressing stable pools after p38α and p38β knockdown was monitored at 24 h and 48 h.

(I) Cell migration and (J) invasion capacity were assessed in 2% FBS and measured at 20 h. The data represent the means ± standard deviation. *, $p<0.05$; **, $p<0.01$; ***, $p< 0.001$; ns, no significant.

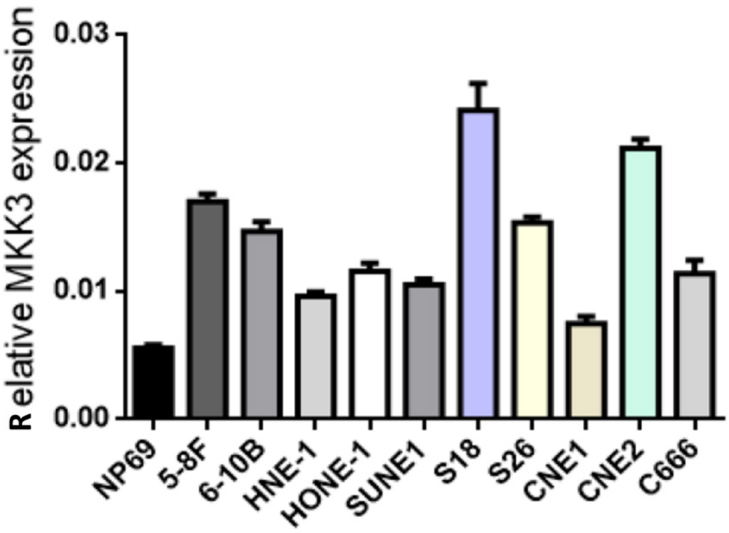

Supplementary Figure S6. MKK3 mRNA expression in NPC cell lines.

MKK3 mRNA expression was determined by qPCR. The data represent the means ± standard deviation.

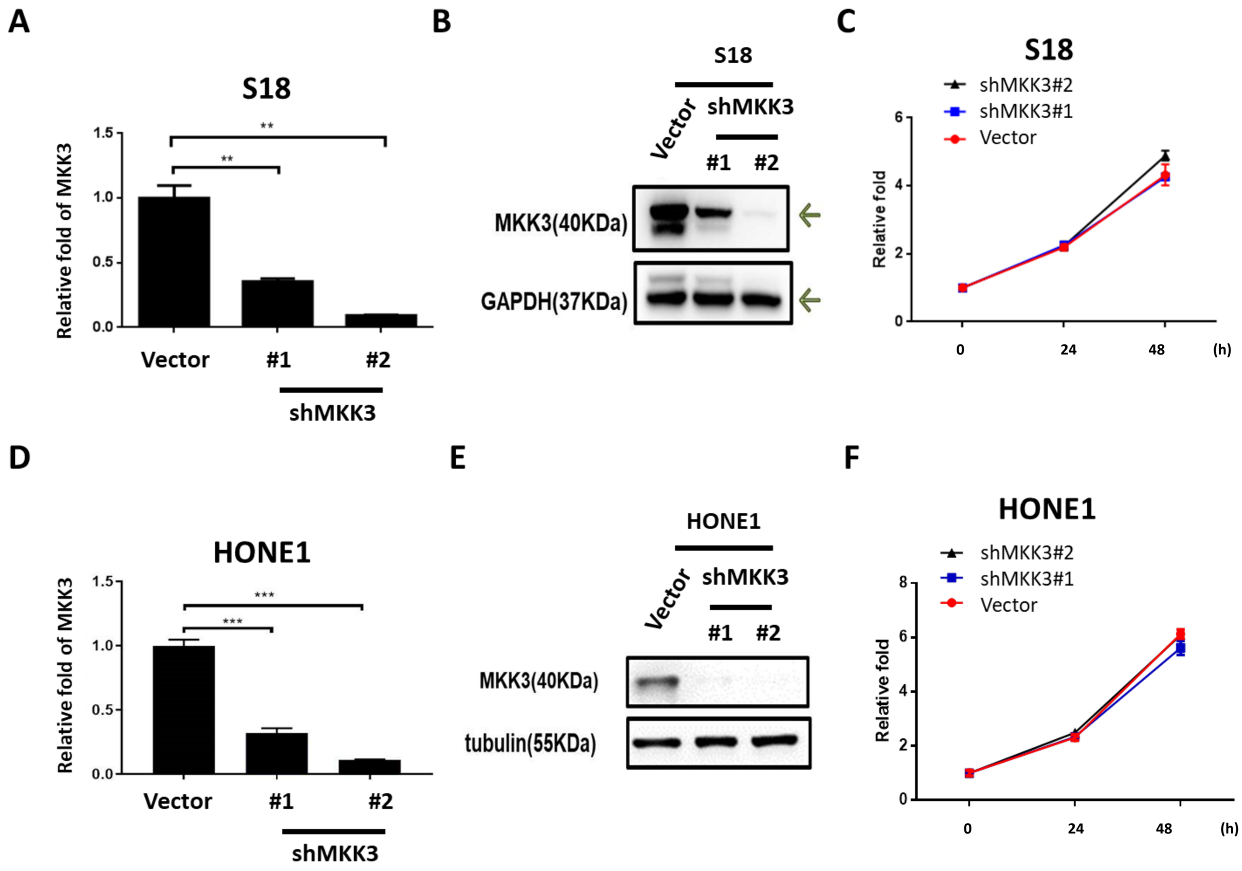

Supplementary Figure S7. Establishment of S18 and HONE1 MKK3-knockdown stable pools.

(A) MKK3 expression was detected by qPCR in S18 MKK3-knockdown stable pools. (B) The MKK3 protein level was reduced by MKK3 knockdown in S18. (C) The cell growth of S18 MKK3-knockdown stable pools was not significantly different when compared with the control group under serum starvation conditions (2% FBS). (D) MKK3 expression was detected by qPCR in HONE1 MKK3-knockdown stable pools. (E) The MKK3 protein level was reduced by MKK3 knockdown in HONE1. (F) The cell growth of HONE1 MKK3-knockdown stable pools was not significantly different when compared with the control group under serum starvation conditions (2% FBS). The data represent the means ± standard deviation. ***p*<0.01; *** *p*<0.0001.

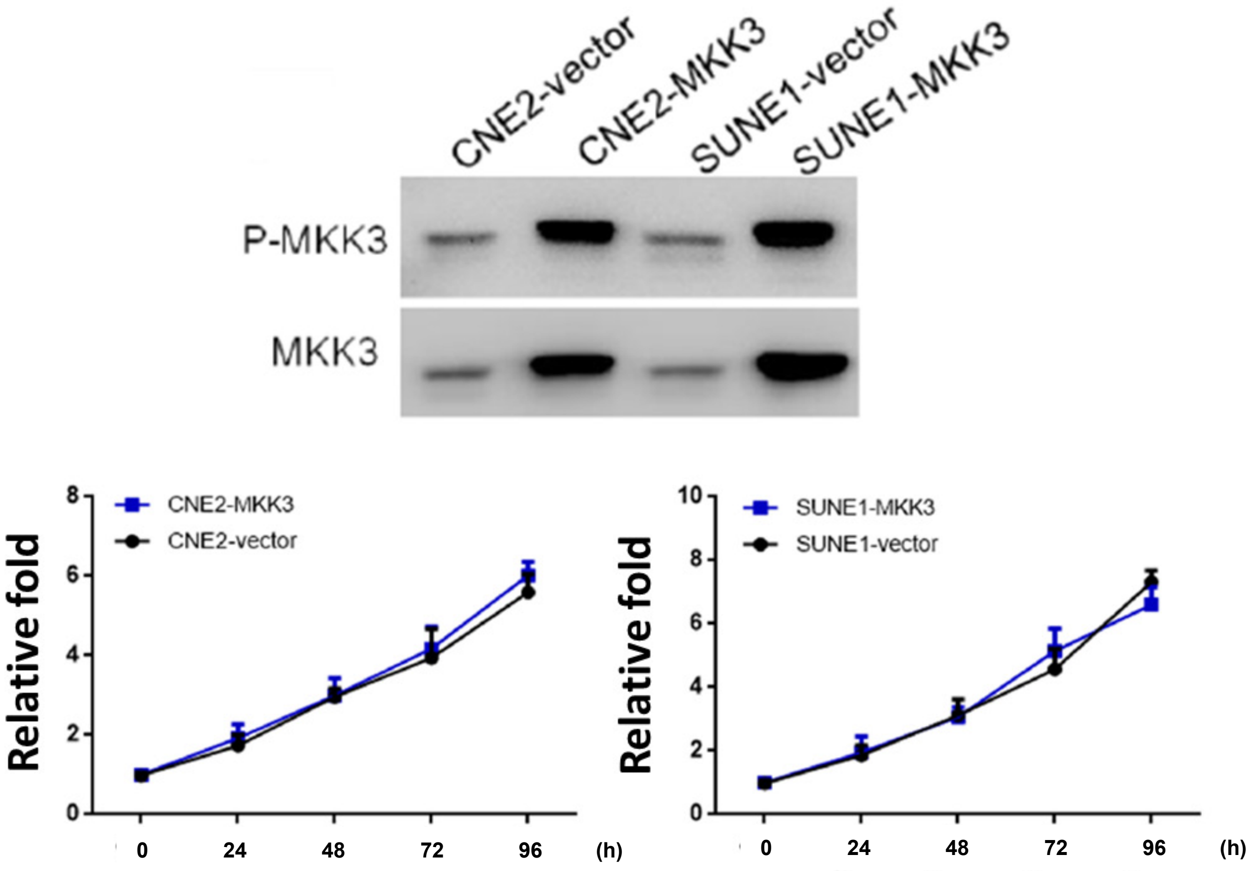

Supplementary Figure S8. Establishment of CNE1 and SUNE1 MKK3-overexpression stable pools.

MKK3 phosphorylation status was promoted by MKK3 overexpression in CNE2 and SUNE1 cells. CNE2 and SUNE1 cell growth was unaffected by MKK3 overexpression.

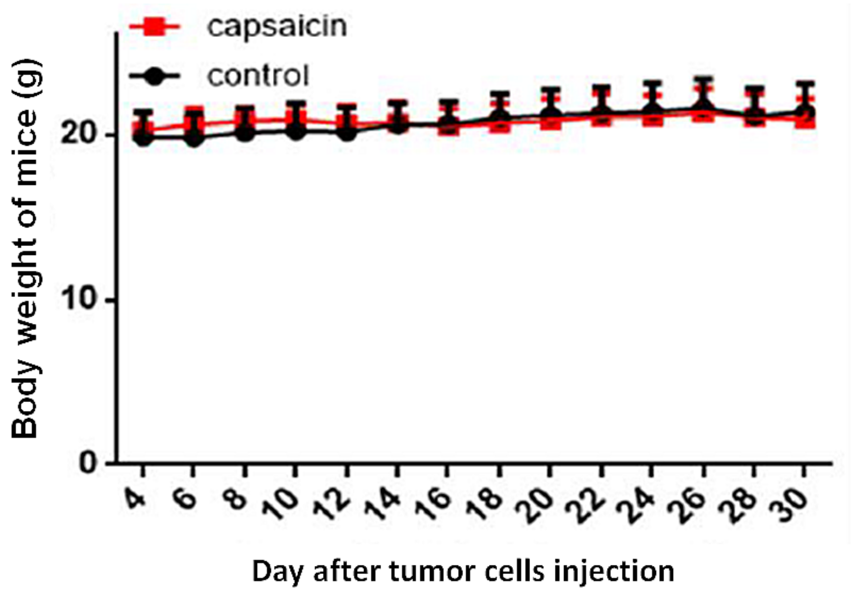

Supplementary Figure S9. Body weight of nude mice in capsaicin-treated and control groups.

The body weights of nude mice were measured every 2 days after injection of tumor cells. There was no significant difference between the capsaicin-treated and control groups.

Supplementary Materials and Methods

Antibodies list

Antibody	Cat. no.	Supplier	Dilution factor
HA-tag	sc-7392	Santa Cruz Biotechnology(Dallas, TX, USA)	1:2000
caspase 3	9665	Cell Signaling Techonlogy (Danvers, MA, USA)	1:1000
cleaved caspase 3	9664	Cell Signaling Techonlogy (Danvers, MA, USA)	1:1000
cleaved caspase 7	8438	Cell Signaling Techonlogy (Danvers, MA, USA)	1:1000
cleaved caspase 9	7237	Cell Signaling Techonlogy (Danvers, MA, USA)	1:1000
cleaved PARP	5625	Cell Signaling Techonlogy (Danvers, MA, USA)	1:1000
cyclin D1	2978	Cell Signaling Techonlogy (Danvers, MA, USA)	1:1000
p27	3686	Cell Signaling Techonlogy (Danvers, MA, USA)	1:1000
MKK3	8535	Cell Signaling Techonlogy (Danvers, MA, USA)	1:1000
phospho-MKK3	12280	Cell Signaling Techonlogy (Danvers, MA, USA)	1:1000
p38	8690	Cell Signaling Techonlogy (Danvers, MA, USA)	1:1000
phospho-p38	4511	Cell Signaling Techonlogy (Danvers, MA, USA)	1:1000
epithelial-mesenchymal transition (EMT) antibody sampler kit	9782	Cell Signaling Techonlogy (Danvers, MA, USA)	1:1000
Flag-tag	F3165	Sigma-Aldrich; Merck	1:2000
β-actin	CW0096A	CoWin BioSciences (Cambridge, MA, USA)	1:2000
α-tubulin	11224-1-AP	ProteinTech Group, Inc. (Rosemont, IL, USA)	1:2000

Oligo-nucleutide list

**Cloning primers**	
MKK3-forward	5’-CGGGGAATTCATGGAGTCGCCGCC-3’
MKK3-reverse	5’-ATAAGAATGCGGCCGCCTACTTATCGTCGTC-3
MKK6-forward	5’-GCTCTAGAATGTCTCAGTCGAAAGGCAAGA-3
MKK6-reverse	5’-CTAGCTAGCTCAGGCGTAGTCGGGGACGTCGTAGGGGTACATGTCTCCAAGAATC-3
**qPCR primers**	
MKK3-forward	5’-TACACTGTCACCTTCTAC-3’
MKK3-reverse	5’-GTCCTCTGGAATTGTCAT-3’
FUK-forward	5’-CAGATTGTGCACTCCCAGGT-3’
FUK-reverse	5’-CTGTATCCAGGCCAGTCACC-3’

GAPDH-forward	5’-AGGTGAAGGTCGGAGTCAAC-3’
GAPDH-reverse	5’-AGTTGAGGTCAATGAAGGGG-3’
**Oligo-nucleutide for knockdown**	
shMKK3-#1-Top	5’-GCACGGTCGACTGTTTCTAC-3’
shMKK3-#1-Bottom	5’-GTAGAAACAGTCGACCGTGC-3’
shMKK3-#2-Top	5’-GCTTCTACACTGTCACCTTCT-3’
shMKK3-#2-Bottom	5’-AGAAGGTGACAGTGTAGAAGC-3’
shFUK-#1-Top	5’-GGATCCTCATTCTGCACATGG-3’
shFUK-#1-Bottom	5’-CCATGTGCAGAATGAGGATCC-3’
shFUK-#2-Top	5’-GCTGTCTGTTCCTGCAAATCC-3’
shFUK-#2-Bottom	5’-GGATTTGCAGGAACAGACAGC-3’
shp38α-#1-Top	5’-CCGGGGGCAGATCTGAACAACATTGCTCGAGCAATGTTGTTCAGATCT GCCCTTTTTG-3’
shp38α-#1-Bottom	5’-AATTCAAAAAGGGCAGATCTGAACAACATTGCTCGAGCAATGTTGTTCA GATCTGCCC-3’
shp38α-#2-Top	5’-CCGGGGTCAGTGGGATGCATAATGGCTCGAGCCATTATGCATCCCACT GACCTTTTTG-3’
shp38α-#2-Bottom	5’-AATTCAAAAAGGTCAGTGGGATGCATAATGGCTCGAGCCATTATGCATC CCACTGACC-3’
shp38β-#1-Top	5’-CCGGGAGCGACGAGCACGTTCAATTCTCGAGAATTGAACGTGCTCGTC GCTCTTTTTG-3’
shp38β-#1-Bottom	5’-AATTCAAAAAGAGCGACGAGCACGTTCAATTCTCGAGAATTGAACGTGC TCGTCGCTC-3’
shp38β-#2-Top	5’-CCGGGCATTACAACCAAACAGTGGACTCGAGTCCACTGTTTGGTTGTA ATGCTTTTTG-3’
shp38β-#2-Bottom	5’-AATTCAAAAAGCATTACAACCAAACAGTGGACTCGAGTCCACTGTTTGG TTGTAATGC-3’
shp38β-#3-Top	5’-CCGGGCCATATGATGAGAGCGTTGACTCGAGTCAACGCTCTCATCATA TGGCTTTTTG-3’
shp38β-#3-Bottom	5’-AATTCAAAAAGCCATATGATGAGAGCGTTGACTCGAGTCAACGCTCTCA TCATATGGC-3’
